# Grhl2 Determines the Epithelial Phenotype of Breast Cancers and Promotes Tumor Progression

**DOI:** 10.1371/journal.pone.0050781

**Published:** 2012-12-17

**Authors:** Xiaoyu Xiang, ZhongBin Deng, Xiaoying Zhuang, Songwen Ju, Jingyao Mu, Hong Jiang, Lifeng Zhang, Jun Yan, Donald Miller, Huang-Ge Zhang

**Affiliations:** 1 Louisville Veterans Administration Medical Center, Louisville, Kentucky, United States of America; 2 Brown Cancer Center, Department of Microbiology & Immunology, University of Louisville, Louisville, Kentucky, United States of America; 3 Brown Cancer Center, Department of Medicine, University of Louisville, Louisville, Kentucky, United States of America; Wayne State University School of Medicine, United States of America

## Abstract

Until now the essential transcription factor that determines the epithelial phenotype of breast cancer has not been identified and its role in epithelial-to-mesenchymal transition (EMT) and tumor progression remain unclear. Here, by analyzing large expression profiles of human breast cancer cells, we found an extraordinary correlation between the expression of Grainyhead transcription factor Grhl2 and epithelial marker E-cadherin. Knockdown of Grhl2 expression by shRNA in human mammary epithelial cell MCF10A leads to down-regulation of E-cadherin and EMT. Grhl2 is down-regulated in disseminated cancer cells that have undergone EMT, and over-expression of Grhl2 is sufficient to induce epithelial gene expression. Large clinical datasets reveal that expression of Grhl2 is significantly associated with poor relapse free survival and increased risk of metastasis in breast cancer patients. In mouse models, over-expression of Grhl2 significantly promotes tumor growth and metastasis. Further testing of several Grhl2 regulated genes leads to the same conclusions that the tumorigenic and metastatic potentials of tumor cells are linked to epithelial phenotype but not mesenchymal phenotype. In conclusion, our findings indicate that Grhl2 plays an essential role in the determination of epithelial phenotype of breast cancers, EMT and tumor progression.

## Introduction

Epithelial-to-mesenchymal transition (EMT) has been demonstrated to play a critical role during tumor metastasis [1], which generates cells with migratory and invasive properties that are able to disseminate to distant organs [1], [2]. The molecular mechanisms of EMT during tumor progression are not fully understood. EMT is defined as a series of changes, including cell morphology transformation from cobble-stone like epithelial to spindle fibroblastic-like morphology, loss of epithelial markers (E-cadherin) with concurrent gaining of mesenchymal markers (vimentin and N-cadherin), disruption of cell-cell contacts and loss of epithelial integrity with acquisition of migratory and invasive ability [3]. During the EMT process, transcription factors play crucial roles. Many signaling pathways that trigger cancer cells to undergo EMT converge at a group of transcription factors, including Snai1, Snai2, Zeb1, Zeb2, and Twist1 [4], [5]. These transcription factors suppress E-cadherin and other epithelial specific genes directly or indirectly.

Here, we postulate that there is an epithelial specific transcription factor that determines epithelial phenotype of breast cancer cells and loss of its expression during tumor progression contributes to loss of the epithelial phenotype and leads to epithelial-to-mesenchymal transition. In our previous study [6], we found approximately 3 weeks after 4T1 cells were injected into mammary fat pads of syngeneic mice, the 4T1 cells could be recovered from the lung. These lung recovered 4T1 cells had undergone EMT. Among the genes down-regulated significantly in these disseminated 4T1 cells, Grainyhead transcription factor, Grhl2, became the most attractive candidate. Grhl2 belongs to the Grainyhead transcription factor family that plays an evolutionarily conserved role in regulating epithelial cell differentiation from Drosophila to mammals [7]–[9]. In this study, we demonstrate that Grhl2 is the epithelial specific transcription factor that determines the epithelial phenotype of breast cancers, and plays a critical role in tumor progression.

## Results

### *Grhl2* is expressed specifically in epithelial cells and co-regulated tightly with *E-cadherin* in human breast cancers

4T1 breast tumor is a classic mouse model for studying the molecular mechanisms of metastasis. After injection into the mammary fat pad, 4T1 tumors spontaneously metastasize in a short period to distant organs, such as lung. 4T1 cancer cells can be recovered from lung about three weeks after injecting the tumor. These lung recovered 4T1 cells had a spindle-like mesenchymal morphology (Figure S1A). Further analyses revealed that these lung recovered cells had a loss of E-cadherin expression and other epithelial specific genes (Figure S1B) [6], indicating that these disseminated cancer cells had undergone epithelial-to-mesenchymal transition (EMT). *Grhl2* was one of the genes that were down-regulated significantly in 4T1 cells that had undergone EMT (Figure S1C, S1D). Grhl2 plays an evolutionarily conserved role in regulating epithelial cell differentiation [9], [10], suggesting Grhl2 could be the epithelial specific transcription factor.

To investigate if Grhl2 is associated with the epithelial phenotype in human breast cancers, we analyzed two microarray datasets of human breast cancer cell lines. We used the PAM305 breast cancer subtype gene classifier [11] to identify molecular subtype. We used a molecular definition of epithelial cells (*Cdh1*, *Cldn3*, *Cldn4*, *Cldn7*, *Tjp2*, *Jup* and *CD24*) and mesenchymal cells (*Cdh2*, *Vim* and *Zeb1*) to distinguish epithelial cells and mesenchymal cells [3], [12], [13].

**Figure 1:**
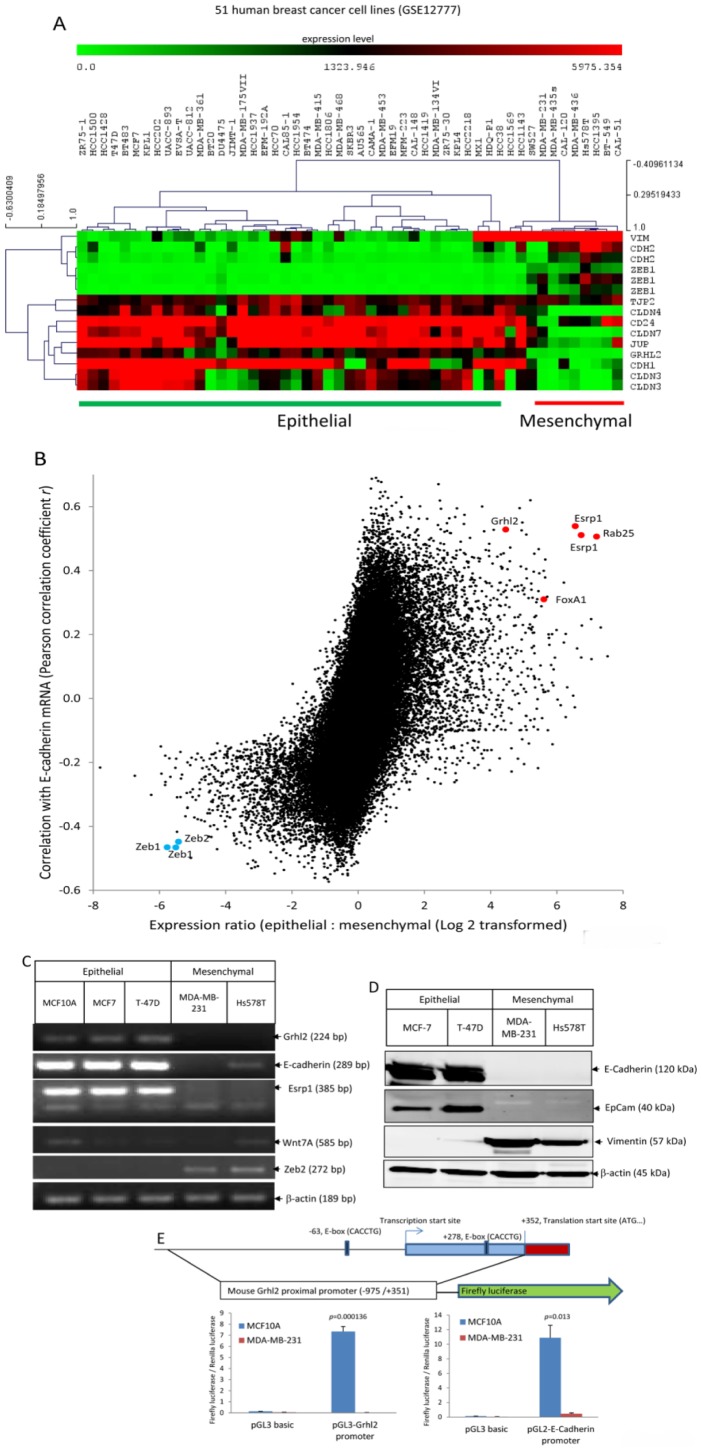
*Grhl2* is specifically expressed in epithelial cells and tightly co-regulated with *E-cadherin* in human breast cancer cells. (A) According to the molecular definition of epithelial and mesenchymal breast cancer cells [1], [12], [13], the following genes were set as epithelial markers: *Cdh1*, *Cldn3*, *Cldn4*, *Cldn7*, *Tjp2*, *Jup*, and *CD24*; these genes were set as markers for mesenchymal cells: *Cdh2*, *Vim* and *Zeb1*. Hierarchical clustering (HCL) of 51 human breast cancer cells (Dataset GSE12777) based on transcription levels of epithelial and mesenchymal markers readily separated these cells into two groups: an epithelial group and a mesenchymal group. *Grhl2* was a positive significant gene of epithelial cells and a negative significant gene of mesenchymal cells (false discovery rate <5%, two-class significance analysis of microarrays (SAM)). *Grhl2* was specifically expressed in epithelial cells with extraordinarily low *p*-values (*p* = 1.27558E-07, two tailed student *t* tests). Red and green squares correspond to high and low mRNA levels, respectively. (B) Microarray based screen for epithelial-specific genes co-regulated with E-cadherin in human breast cancer cells (Dataset GSE12777). All probes representing 54,675 transcripts were analyzed for differential expression between epithelial and mesenchymal cells and Pearson correlation coefficient *r* was calculated between each transcript and E-cadherin across all microarrays. Some of the transcripts that were highly expressed in epithelial cells and co-regulated with E-cadherin are labeled in Red, and some of the transcripts that were highly expressed in mesenchymal and negatively co-regulated with E-cadherin are labeled in Blue. (C) Examination of *Grhl2, E-cadherin, Esrp1, Zeb2* and Wnt7A expression in human mammary epithelial cells and breast cancer cells by RT-PCR. (D) Expression of *E-cadherin*, *Epcam*, and *vimentin* in human breast cancer cell lines was examined by western blotting. (E) *Grhl2* promoter activity in epithelial and mesenchymal breast cells. Upper panel: A schematic map of mouse *Grhl2* proximal promoter region and luciferase reporter. Bottom panel: Luciferase reporters containing mouse *Grhl2* proximal promoter, E-cadherin promoter, or empty vector (pGL3basic) were transiently transfected into MCF10A and MDA-MB-231 cells with a Renilla luciferase vector for normalization. Dual luciferase activities were measured 48 hours post transfection. Data represent one of three independent experiments. Error bars represent mean ± SEM of duplicated experiments. *p* values were calculated by two tailed student *t* test.

Dataset GSE12777 contained microarray data of 51 human breast cancer cells [14]. Hierarchical clustering (HCL) of cells based on transcription levels of the epithelial and mesenchymal markers readily separated these cells into two groups: an epithelial group and a mesenchymal group (Figure 1A). We performed two-class significance analysis of microarrays (SAM) to identify epithelial specific genes (false discovery rate <5%). *Grhl2* was specifically expressed in epithelial cells with an extraordinarily low *p*-value (*p* = 1.27558E-07, two tailed student *t* tests), suggesting a strong association with epithelial phenotypes. In addition, we calculated standard Pearson correlation coefficients of all transcripts (54675 transcripts) on the microarray with the epithelial marker E-cadherin, or with the mesechymal marker vimentin. *Grhl2* was one of a few genes that was over-expressed in epithelial cells and showed a strong correlation with *E-cadherin* (*r* = 0.53) (Figure 1B), and negative correlation with *vimentin* (*r* = −0.57) (Figure S2A). Furthermore, among the 3000 transcripts that were annotated as transcription factors, DNA binding proteins or RNA binding proteins, *Grhl2* was one of few that displayed the strongest correlation with *E-cahderin* and the highest expression ratio between epithelial cells and mesenchymal cells (Figure S2B). Using the PAM305 breast cancer subtype gene classifier [11], these cell lines can be separated into three groups, a luminal group expressing luminal cell markers, a basal A group expressing myoepithelial cell markers, and a basal B group expressing mesenchymal markers. Grhl2 is expressed in both luminal group and basal A group, but not basal B group (Figure S2C). Dataset GSE16795 contains microarray data of 39 human breast cancer cell lines [15]. Results from Dataset GSE16795 also revealed that *Grhl2* was specifically expressed in epithelial cells (*p* = 3.9E-8, two tailed student *t* tests) (Figure S2D). Above all, these data indicate that *Grhl2* is specifically expressed in epithelial cells and tightly correlated with *E-cadherin* in human breast cancer cells.

To confirm the microarray data, RT-PCR was performed on some breast cancer cells. Results from RT-PCR clearly showed that transcripts of *Grhl2* were abundant in epithelial cells, including MCF10A, MCF7, and T-47D, but was undetectable in mesenchymal breast cells, including MDA-MB-231 and Hs578T (Figure 1C). The RT-PCR data was further confirmed by western blot analysis (Figure 1D).

To further confirm the results, we analyzed the *Grhl2* promoter activities in human epithelial and mesenchymal breast cell lines by using a luciferase reporter. Examination of the genome location of mouse *Grhl2* in the vicinity of the transcription start site using the UCSC genome browser indicates conserved CpG islands lying within this region (Figure 1E, top panel), suggesting a promoter region. In addition, this region is highly conserved across human, mouse and rat. The DNA sequence of mouse in this region (−183/+320) shares 94% sequence identity with the human sequence, suggesting that regulation of *Grhl2* in human and mouse is probably conserved also. We cloned the proximal promoter of mouse *Grhl2* and placed it upstream of a firefly luciferase reporter gene in a pGL3 basic vector. *Grhl2* promoter driving luciferase activities was detected in MCF10A cells but not in MDA-MB-231 cells (*p* = 0.000136, two-tailed student *t* tests) (Figure 1E, bottom panel). As a control, E-cadherin promoter driving luciferase activity was assayed in these two cell lines also. E-cadherin promoter driving luciferase activities were detected only in MCF10A cells, but not in MDA-MB-231 cells (*p* = 0.013, two-tailed student *t* tests) (Figure 1E, right bottom panel). In conclusion, our data generated by analyzing human breast cancer microarray data, RT-PCR, and promoter driving luciferase reporters indicate that *Grhl2* is specifically expressed in epithelial cells and tightly co-regulated with E-cadherin, suggesting that Grhl2 is the essential transcription factor that determines the epithelial phenotype of breast cancer cells.

### *Grhl2* determines the epithelial phenotype of mammary epithelial cells

**Figure 2:**
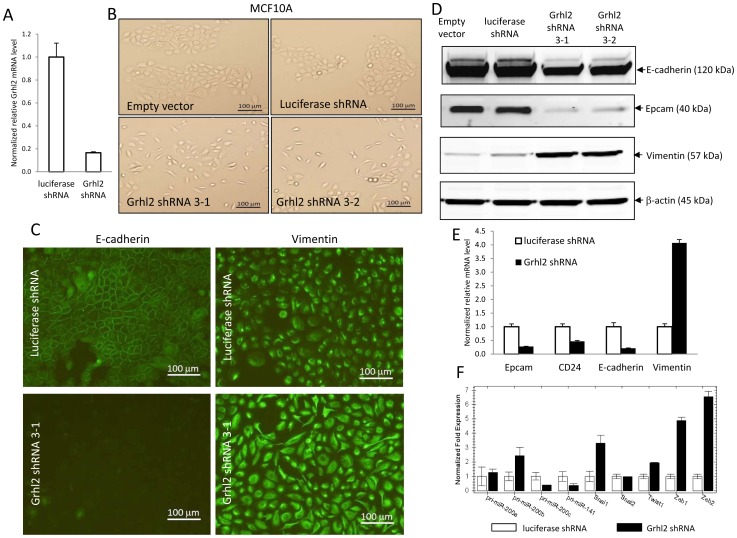
Grhl2 determines the epithelial phenotype of mammary epithelial cells. (A) shRNA constructs against firefly luciferase (as a control ) and *Grhl2* were introduced into MCF10A cells by retroviral transfection. Efficient knockdown of *Grhl2* by shRNA in MCF10A cells was verified by quantitative realtime PCR. Error bars represent mean ± SEM of duplicated experiments. (B) *Grhl2* knockdown in MCF10A cells led to cell morphology changes from cobble-stone like epithelial to spindle-like mesenchymal morphology (10× objective). Grhl2 shRNA3-1 and Grhl2 shRNA3-2 were two replicas of *Grhl2* shRNA retroviral transfected MCF10A cells. (C) Immunofluorescent staining of E-cadherin and vimentin in MCF10A luciferase ShRNA and MCF10A Grhl2 shRNA cells (10× objective). One example representative of three independent experiments is shown. (D) Expression of epithelial and mesenchymal markers was assayed by western blotting. One example representative of three independent experiments is shown. (E) Relative transcription levels of epithelial and mesenchymal markers were assayed by quantitative realtime PCR. Error bars represent mean ± SEM of duplicate experiments. (F) Knock down of *Grhl2* resulted in down-regulation of microRNA-200c∼141 cluster and up-regulation of EMT inducers. Relative expression levels of primary microRNA-200C∼141 cluster, primary microRNA-200b∼200a cluster, and EMT inducers were measured by quantitative realtime PCR. Error bars represent mean ± SEM of duplicate experiments.

Next, we tested if Grhl2 is the essential transcription factor that determines the epithelial phenotype of mammary epithelial cells. shRNA vectors against firefly *luciferase* (as a control) and *Grhl2* were introduced into the human mammary epithelial cell line MCF10A by retroviral transfection. Efficient knockdown of *Grhl2* mRNA by shRNA was verified by quantitative real-time PCR (Figure 2A). In comparison with empty vector or the luciferase shRNA vector – infected cells, knockdown of *Grhl2* in MCF10A cells led to significant cell morphology changes, with transformation of the cobble-stone like epithelial morphology to an elongated fibroblast-like mesenchymal morphology (Figure 2B). To confirm that the phenotype changes observed in these cells was due to loss of the epithelial phenotype, we performed immunofluorescent staining of epithelial and mesenchymal markers. The immunofluorescence images clearly showed drastically reduced endogenous E-cadherin protein levels, and more importantly disruption of cell-cell border E-cadherin staining in *Grhl2* knockdown cells (Figure 2C, left panel), indicating a breakdown of cell-cell junctions. Furthermore, staining of the mesenchymal marker vimentin was increased significantly (Figure 2C, right panel), indicating that knockdown of *Grhl2* led to MCF10A cells to undergo epithelial-to-mesenchymal transition (EMT). The EMT phenotype was further confirmed by expression of characteristic molecular markers using western blotting (Figure 2D) and realtime PCR (Figure 2E, 2F). In *Grhl2* knockdown MCF10A cells, epithelial markers *E-cadherin*, *Epcam*, and *CD24* were down-regulated, while the mesenchymal marker vimentin was up-regulated dramatically (Figure 2D, 2E). EMT inducers, including *Snai1*, *Twist1*, *Zeb1* and *Zeb2*, were up-regulated in *Grhl2* knockdown MCF10A cells while expression levels of primary microRNA of miR-200c∼141 cluster whose products are negative regulators of Zeb1 and Zeb2, was decreased (Figure 2F). Collectively, these data indicate that Grhl2 is essential for mammary epithelial cells to maintain the epithelial phenotype. Knockdown of *Grhl2* leads to a loss of epithelial morphology, epithelial markers and induction of EMT. Our data showed that 4T1 cells recovered from lung did not express Grhl2 and had undergone EMT. These data suggest that loss of Grhl2 during tumor progression could contribute to loss of the epithelial phenotype and promote EMT.

### Over-expression of *Grhl2* induces epithelial gene expression and promotes tumor growth and metastasis

Our data showed that Grhl2 determines the epithelial phenotypes of breast cancers. Next, we tested if over-expression of Grhl2 was able to inhibit EMT during tumor progression and block tumor metastasis. We used the 4T1 model to answer this question, in which disseminated cancer cells had undergone EMT and did not express Grhl2 (Figure S1A, S1B, S1C).

**Figure 3:**
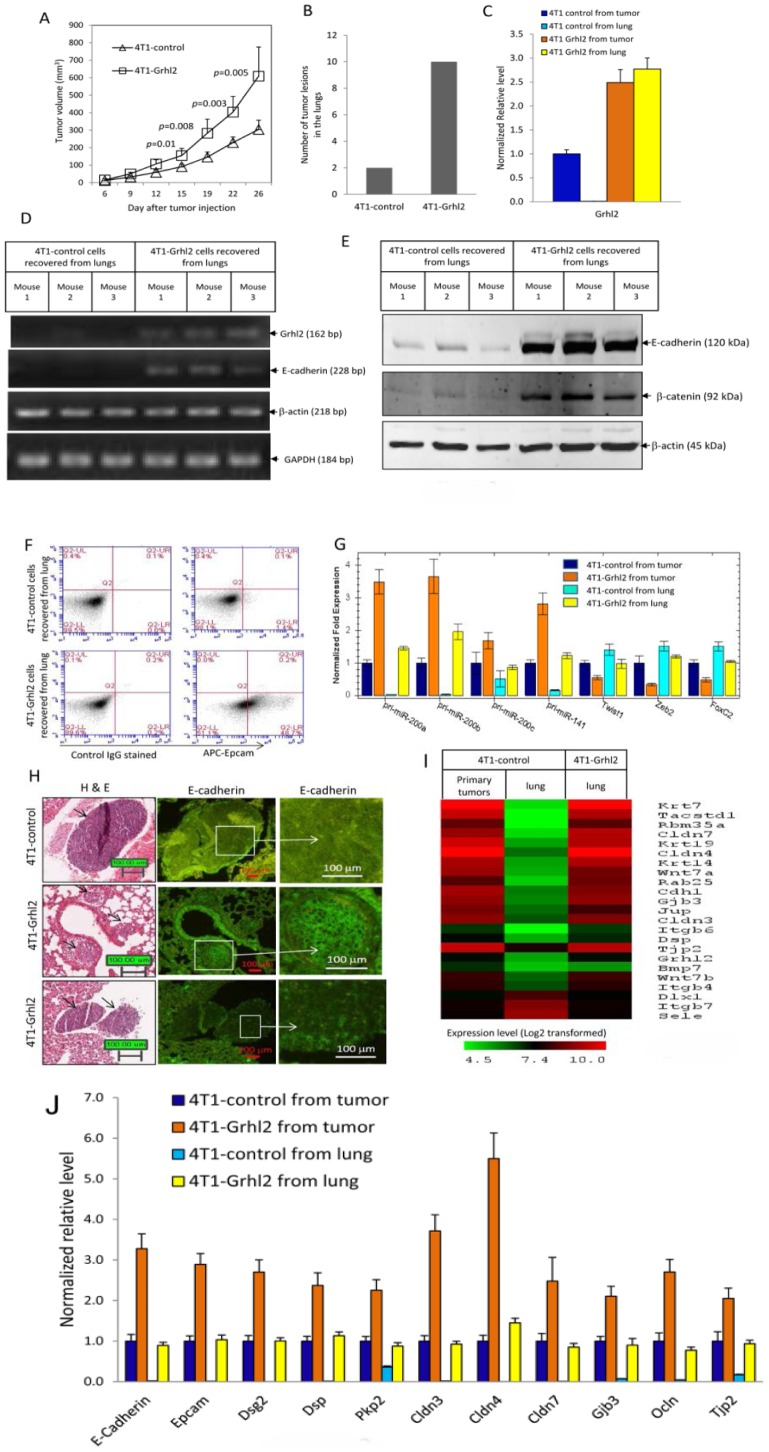
Stable expression of Grhl2 induces epithelial gene expression and promotes tumor growth and metastasis. (A) Full length open reading frame of mouse Grhl2 was cloned from a cDNA pool derived from 4T1 cells and was verified by sequencing. Grhl2 was introduced into 4T1 cells by lentiviral transfection. One week post virus infection, GFP^+^ cells were sorted. 6-week-old female BALB/c mice were injected with 4T1-control or 4T1-Grhl2 cells (1.0×10^5^ cells in 100 µl PBS for each mouse; 4T1-control, n = 18 mice; 4T1-Grhl2, n = 18 mice). Tumor size was measured twice weekly. Data represents one of four independent experiments. Each data point represents the mean ± SEM of five primary tumors. (B) Lung metastasis was examined by hematoxylin and eosin (HE) staining of paraffin sections of fixed lung tissue. The total number of metastatic tumor lesions observed in lungs from each group was counted. Data represent one of four independent experiments (n = 18 mice). (C) Relative transcription levels of Grhl2 in recovered 4T1 cells were measured by quantitative realtime PCR. Error bars represent the mean ± SEM of duplicate experiments. Data represent one of four independent experiments. (D) Grhl2 and E-cadherin mRNA expression in cancer cells recovered from lungs of individual mice carrying either 4T1-control or 4T1-Grhl2 tumors were analyzed by RT-PCR. Data represent one of four independent experiments. RNAs isolated from cells recovered from lungs of three different mice from each group were assayed. (E) E-cadherin protein expression in 4T1 cells recovered from lungs of individual mice was examined by western blotting. Data represent one of four independent experiments. Proteins extracted from cells recovered from lungs of three different mice from each groups were assayed. (F) 4T1 cells recovered from lungs of individual mice were stained with APC labeled anti-Epcam antibody and analyzed by flow cytometry. Data represent one of four independent experiments (n = 18). (G) Relative transcription levels of EMT regulators in recovered 4T1 cells were analyzed by quantitative realtime PCR. Error bars represent the mean ± SEM of duplicate experiments. Data represent one of four independent experiments (n = 18). (H) H&E staining (Left panels) and E-cadherin immunofluorescent staining (middle and right panels) of paraffin sections of fixed lung tissue. Arrows indicate metastatic tumors. Boxed area is further enlarged in the right panels. E-cadherin is located at cell-cell borders. (I) Microarray gene expression profiling 4T1-control cells recovered from primary tumors and lungs, and 4T1-Grhl2 cells recovered from lungs. Heatmap depicts genes that were down-regulated in 4T1-control cells recovered from lung with the highest fold changes are also the genes that are up-regulated in 4T1-Grhl2 cells recovered from lung with the highest fold changes. (J) Confirmation of some of the microarray data by quantitative realtime PCR. Error bars represent the mean ± SEM of duplicate experiments.

4T1 cells were transfected with lentiviral constructs expressing *Grhl2* or empty vector. A pool of sorted GFP^+^ cells was used for the *in vivo* studies, thus avoiding clonal selection bias. In *Grhl2* over-expressing cells, the transcription level of *Grhl2* was four times that of the control cells as measured by quantitative realtime PCR (Figure S3A). Over-expression of *Grhl2* caused an up-regulation of primary transcripts of microRNA-200b∼200a∼429 cluster and Epcam, and down-regulation of *Zeb2* (Figure S3A, S3B). Surprisingly, tumors formed by 4T1-Grhl2 cells were significantly larger (*p* = 0.005, two tailed student *t* tests) (Figure 3A), and more metastatic tumors were formed in the lungs than 4T1-control cells (Figure 3B) (18 mice in each group, four independent experiments).

Using RT-PCR, we confirmed high levels of *Grhl2* transcripts in 4T1-Grhl2 cells recovered from primary tumors and lungs (Figure 3C, 3D). Consistent with our previous data, 4T1-control cells recovered from lungs had undergone EMT, and did not express *Grhl2*, *E-cadherin* and *Epcam* (Figure 3D, 3E, 3F, 3G). In 4T1-Grhl2 cells recovered from lungs, high levels of *E-cadherin*, *Epcam* and *miR-200s* expression were detected (Figure 3D, 3E, 3F, 3G). Immunofluorescent staining of fixed lung sections also confirmed E-cadherin staining in the metastatic tumors formed by 4T1-Grhl2 cells, but not in metastatic tumors originating from 4T1-control cells (Figure 3H). Together, these data indicate that stable expression of Grhl2 induces E-cadherin expression in 4T1 cells at metastatic sites. Surprisingly, these data also show that stable expression of Grhl2 promotes tumor growth and metastasis.

To identify downstream target genes of Grhl2, we performed microarray gene expression profiling. 4T1-control cells recovered from lung had undergone EMT and did not express Grhl2. Compare to 4T1-control cells recovered from primary tumors 260 transcripts were down-regulated and 33 transcripts were up-regulated (<2.0 fold) in the 4T1-control cells recovered from the lungs (>2.0 fold) (Table S1). Among the 260 transcripts that were down-regulated in 4T1-control cells from the lung, 133 transcripts were up-regulated (>2.0 fold) in 4T1-Grhl2 cells recovered from the lung. In addition, genes that were down-regulated in the 4T1-control cells from the lung that had the highest fold changes were also the genes that also were up-regulated in 4T1-Grhl2 cells with the highest fold changes (Figure 3I, Table S1), suggesting these genes are directly or indirectly regulated by Grhl2. Genes regulated by Grhl2 cover a broad range of epithelial characteristics, including cell adhesion (*Cdh1*), tight junction (*Cldn7*, *Cldn4*, *Cldn3*, *Ocln* and *Tjp2*), desmosomes (*Dsp*, *Jup*, *Pkp2* and *Dsg2*), gap junction (*Gjb3*), cytokeratine (*Krt7*, *Krt8*, *Krt14*, *Krt17* and *Krt19*), Integrin (*Itgb6*, *Itgb4* and *Itgb2*), Wnt ligands (*Wnt7A, Wnt7B*) and epithelial specific splicing factor (*Esrp1*). Some of the data from microarrays were further confirmed by quantitative realtime PCR (Figure 3J). Collectively, these data reveal that 51.5% (133/260) of genes down-regulated in 4T1 cells that had undergone EMT can be reversed by Grhl2, indicating a critical role of Grhl2 in maintaining the epithelial phenotype.

### Wnt7A is regulated by Grhl2, and over-expression of Wnt7A phenotypically mimics Grhl2 in promoting epithelial gene expression and tumor progression

**Figure 4:**
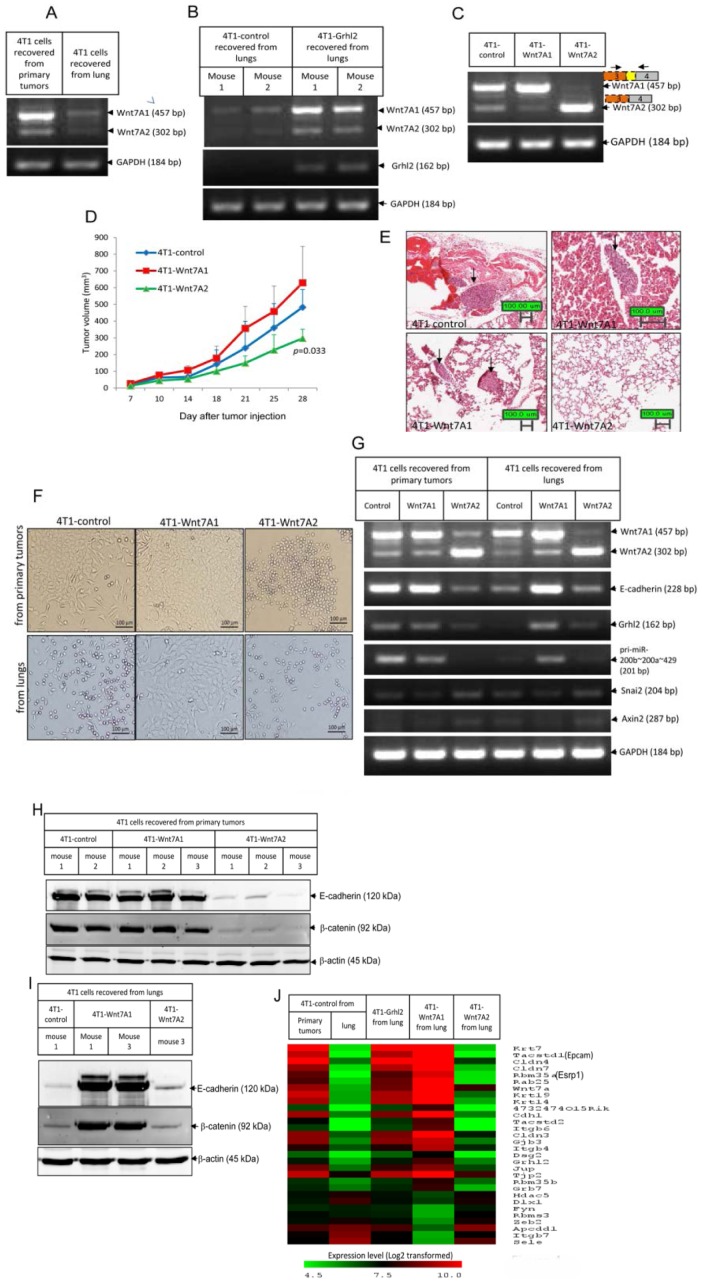
Wnt7A is regulated by Grhl2, and over-expression of Wnt7A phenotypically mimics Grhl2 in promoting epithelial gene expression and promoting tumor progression. (A) Wnt7A was down-regulated in 4T1 cells recovered from lung that had undergone EMT. Expression of Wnt7A in 4T1 cells recovered from primary tumors and lungs were examined by RT-PCR. Data represent one of four independent experiments (n = 18). (B) Forced expression of Grhl2 reversed Wnt7A expression in 4T1 cells recovered from lung. Wnt7A mRNA expression in cancer cells recovered from lungs of individual mice carrying either 4T1-control or 4T1-Grhl2 tumors were analyzed by RT-PCR. Data represent RNAs isolated from cells recovered from lungs of two different mice from each group (n = 18). (C) Constructing 4T1 cell lines stably expressing Wnt7A1 and Wnt7A2. Wnt7A1 and Wnt7A2 were introduced into 4T1 cells by lentiviral infection. One week after virus infection GFP^+^ cells were sorted. Gene expressions in 4T1-control, 4T1-Wnt7A1 and 4T1-Wnt7A2 cells were analyzed by RT-PCR. On the top are schematics of gene structures of Wnt7A1 and Wnt7A2. (D) Growth curves of tumors formed by 4T1-control, 4T1-Wnt7A1 and 4T1-Wnt7A2 in wild type BALB/c mice. 6-week-old female BALB/c mice were injected with 4T1-control, 4T1-Wnt7A1, or 4T1-Wnt7A2 cells (1.0×10^5^ cells in 100 ul PBS for each mouse; 4T1-control, n = 6 mice; 4T1-Wnt7A1, n = 7 mice; 4T1-Wnt7A2, n = 5 mice). Tumor size was measured twice a week. Each data point represents the mean ± SD of four primary tumors. Data represent one of two independent experiments. (E) Lung metastasis was examined by hematoxylin and eosin (HE) staining of paraffin sections of fixed lung tissues (4T1-control, n = 6 mice; 4T1-Wnt7A1, n = 7 mice; 4T1-Wnt7A2, n = 5 mice). Arrows indicate metastatic tumors. Data represent one of two independent experiments. (F) Representative micrographs of 4T1-control, 4T1-Wnt7A1 and 4T1-Wnt7A2 cells recovered from primary tumors and lungs. (G) Representative gene expressions in recovered 4T1 cells analyzed by RT-PCR (4T1-control, n = 6 mice; 4T1-Wnt7A1, n = 7 mice; 4T1-Wnt7A2, n = 5mice). (H) E-Cadherin and β-catenin protein expression in cancer cells recovered from primary tumors of individual mice carrying 4T1-control, 4T1-Wnt7A1 or 4T1-Wnt7A2 tumors examined by Western blotting. (I) E-Cadherin and β-catenin protein expression in cancer cells recovered from lungs of individual mice carrying 4T1-control, 4T1-Wnt7A1 or 4T1-Wnt7A2 tumors examined by Western blotting. (J) Heatmap showing genes up-regulated and down-regulated, with the highest fold changes by Wnt7A1 which overlapped with genes regulated by Grhl2.

Our data show that Grhl2 promotes epithelial gene expression and also promotes tumor growth and metastasis. Next, we tested whether Grhl2 mediated promotion of tumor growth and metastasis is through its regulated genes. Among them, Wnt7A is a very attractive candidate. Using RT-PCR, we confirmed that Wnt7A was down-regulated significantly in 4T1 cells that had undergone EMT (Figure 4A), and stable expression of Grhl2 up-regulated Wnt7A expression in 4T1 cells recovered from the lung (Figure 4B).

Using specific primers to amplify the full length open reading frame of mouse Wnt7A from a cDNA pool derived from 4T1 cells, we obtained two cDNA clones. Sequencing results showed that the longer cDNA shared 100% sequence identity with the reference sequence of mouse Wnt7A (Accession number: NM_009527.3), encoding a protein with 349 amino acids in length. This isoform is designated as Wnt7A isoform 1 or Wnt7A1 (Figure S4A). The shorter cDNA lacked 155 base pairs compared to the reference sequence of mouse Wnt7A, due to using an alternative 5′ splice site inside exon3, which results in introducing an in-frame stop codon immediately after the alternative splicing site. This isoform encodes a truncated Wnt7A protein 148 amino acids in length. This isoform is designated as Wnt7A isoform 2 or Wnt7A2 (Figure S4A, S4B). In 4T1 cells in the epithelial state, Wnt7A1 is the dominant isoform (Figure 4A, 4B). Stable expression of Wnt7A1 in 4T1 cells caused a slight up-regulation of *E-cadherin*, the primary transcript of *miR-200s*, and a down-regulation of *Twist1* and *vimentin*, while stable expression of Wnt7A2 led to down-regulation of *E-cadherin*, *Epcam*, *Grhl2*, and primary transcripts of *miR-200s* (Figure 4C, S4C).

Next, these cells were transplanted into mammary fat pads of female BALB/c mice, and tumor progression was monitored every three days. Tumor growth was significantly delayed in the 4T1-Wnt7A2 group when compared to the other two groups (Figure 4D). At four weeks post tumor implantation, the average size of 4T1-Wnt7A2 tumors was 47% of the size of 4T1-Wnt7A1 tumors, and 62% of the size of 4T1-control tumors making them significantly smaller (*p* = 0.033, two-tailed student *t* test) (Figure 4D). Lung tumor metastatic lesions were observed in mice bearing 4T1-Wnt7A1 tumors and 4T1-control tumors, but not in mice bearing 4T1-Wnt7A2 tumors (Figure 4E). These results indicate that Wnt7A1 promotes tumor growth, while Wnt7A2 inhibits tumor growth and metastasis.

We then examined gene expression in cells recovered from primary tumors and lungs. 4T1-control cells recovered from primary tumors were mainly comprised of epithelial cells, and expressed *E-cadherin*, *Grhl2* and *miR-200*, while 4T1-control cells recovered from lungs were characterized by mesenchymal morphology and did not express *E-cadherin*, *Grhl2* and *miR-200* (Figure 4F, 4G, 4H, 4I). 4T1-Wnt7A1 cells recovered from either primary tumors or lungs were characterized by a cobble-stone like epithelial morphology and expressed high levels of *E-cadherin*, *Grhl2* and *miR-200* (Figure 4F, 4G, 4H, 4I). In contrast, 4T1-Wnt7A2 cells recovered from either primary tumors or lungs had a spindle or round-like mesenchymal morphology and did not express *E-cadherin*, *Grhl2* and *miR-200* (Figure 4F, 4G, 4H, 4I).

We also obtained the gene expression profiles of 4T1-Wnt7A1 and 4T1-Wnt7A2 cells recovered from lungs. Compared to 4T1-control cells recovered from lung, 419 transcripts were up-regulated (>2.0 fold) in 4T1-Wnt7A1 cells recovered from lung (Table S2). Among them, 156 transcripts overlapped with genes up-regulated in 4T1-Grhl2 cells from the lung (>2.0 fold). Genes up-regulated in 4T1-Wnt7A1 cells with the highest fold changes were the same genes that were up-regulated by Grhl2, including *Cdh1*, *Cldn4*, *Cldn7*, *Dsg2*, *Gjb3*, *Tjp2*, *Esrp1*, *Rab25*, and *Epcam* (Figure 4J). These data indicate that Wnt7A1 signaling mediated induction and expression of Grhl2 and other epithelial genes; while Wnt7A2, which encodes a truncated Wnt7A protein, causes in 4T1 cells to undergo EMT probably by competing for the same receptor as Wnt7A1 or perhaps some other mechanism.

### *Esrp1* is regulated by Grhl2 and stable-expression of a variant isoform of *Esrp1* (*Esrp1-V1*) leads to 4T1 cells to undergo EMT and inhibits tumor progression

**Figure 5:**
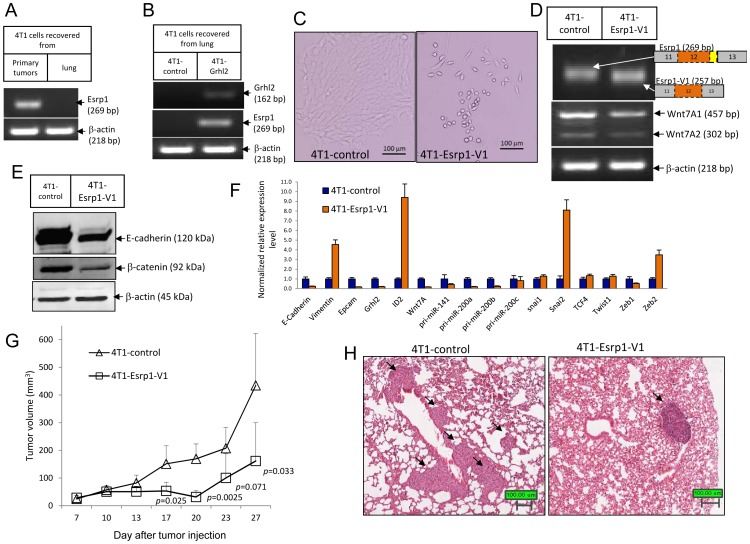
*Esrp1* is regulated by Grhl2, and stable-expression of a variant isoform of *Esrp1* (*Esrp1-V1*) leads 4T1 cells to undergo EMT and inhibits tumor progression. (A) *Esrp1* was down-regulated in 4T1 cells recovered from lung. Esrp1 expression in 4T1 cells recovered from primary tumors and lungs was analyzed by RT-PCR. Data represent one of four independent experiments (n = 18). (B) Stable expression of *Grhl2* reversed *Esrp1* expression in 4T1 cells recovered from lung. Gene expression in 4T1-control and 4T1-Grhl2 cells recovered from lung was analyzed by RT-PCR. Data represent one of four independent experiments (n = 18). (C) Representative micrographs of 4T1-control and 4T1-Esrp1-V1 cells. Stable expression of a variant isoform of Esrp1 (Esrp1-V1) led to transformation of 4T1 cells from cobble-stone like epithelial morphology to spindle-like mesenchymal morphology. Esrp1-V1 was cloned from a cDNA pool derived from 4T1 cells that lacked 12 in frame base pairs compared to the reference sequence of mouse Esrp1 (NM_194055). Esrp1-V1 was introduced into 4T1 cells by lentiviral infection. (D) Validation of Esrp1-V1 expression in 4T1-Esrp1-V1 cells by RT-PCR. On the right are the schematics depicting the gene structures of Esrp1 and Esrp1-V1 based on sequencing results. Esrp1-V1 is 12 base pairs shorter than the dominant transcript of Esrp1 in 4T1 cells due to using an alternative splicing site within exon12, which is why it migrates slightly faster in 2.5% agarose Gels. Expression of Wnt7A1 was down-regulated significantly in 4T1-Esrp1-V1 cells. (E) E-cadherin and β-catenin expression in 4T1-control and 4T1-Esrp1-V1 cells were analyzed by Western blotting. (F) Expression changes of EMT markers in 4T1-Esrp1-V1 cells compared to 4T1-control cells were analyzed by quantitative realtime PCR. Error bars represent the mean ± SEM of duplicate experiments. (G) Growth curves of tumors formed by 4T1-control and 4T1-Esrp1-V1 in wild type BALB/c mice. 6-week-old female BALB/c mice were injected with 4T1-control or 4T1-Esrp1-V1 cells (1.0×10^5^ cells in 100 ul PBS for each mouse; 4T1-control, n = 8 mice; 4T1-Esrp1-V1, n = 9 mice). Tumor size was measured twice weekly. Data represents one of two independent experiments. Each data point represents the mean ± SD of five primary tumors. (H) Lung metastasis was examined by hematoxylin and eosin (HE) staining of paraffin sections of fixed lung tissues (4T1-control, n = 8 mice; 4T1-Esrp1-V1, n = 9 mice). Arrows indicate metastatic tumors.

Our data have shown that Grhl2 and Wnt7A1 promote epithelial gene expression and tumor progression while Wnt7A2 promotes EMT but inhibits tumor progression, suggesting that 4T1 tumor progression *in vivo* might be associated with the epithelial phenotype. Based on these data, we hypothesized that a gene that promotes EMT would inhibit tumor progression. To test this hypothesis, we tested another Grhl2 regulated gene, i.e., Esrp1 an epithelial cell specific splicing factor that controls epithelial specific splicing of its targeted genes [16]. *Esrp1* was down-regulated in 4T1 cells recovered from the lung that had undergone EMT (Figure 5A), correlating with its targeted gene splicing switching from the epithelial to the mesenchymal isoform, including *CD44*, *CTNND1*, *MAP3K7* and *Enah* (Figure S5A). Over-expression of *Grhl2* up-regulates *Esrp1* expression (Figure 3I, 4J, 5B).

We obtained two cDNA clones of mouse Esrp1 from a cDNA pool derived from 4T1 cells. Sequencing results showed that one clone lacked 12 in-frame base pairs compared to the reference sequence of mouse Esrp1 (NM_194055), due to using an alternative 5′ splicing site inside exon12 (Figure S5B). This isoform is designated as Esrp1 variant isoform 1 (Esrp1-V1). Searching EST database revealed that using this alternative splicing site was very common both in human and mouse cells (Figure S5C). Esrp1 contains three conserved RNA recognition domain (RRM) [16]. The four amino acids missing in Esrp1-V1 are not located in any of the RRM domains. Surprisingly, stable expression of Esrp1-V1 in 4T1 cells led to significant cell morphology changes with a transformation from cobble-stone like epithelial morphology to spindle like mesenchymal morphology (Figure 5C, 5D). Expression of epithelial markers, including *E-cadherin*, *Epcam*, *Grhl2*, as well as *Wnt7A*, were significantly down-regulated, while mesenchymal markers, including *vimentin*, *Snai2* and *Zeb2*, were up-regulated in 4T1-Esrp1-V1 cells (Figure 5D, 5E, 5F). Esrp1-V1 also perturbed Esrp1 regulated alternative splicing, with switching of its targeted genes, including *CD44*, *CTNND1*, *MAP3K7* and *Enah*, from the epithelial specific isoform to the mesenchymal specific isoform (Figure S5D). Together, these data indicate that the Esrp1 regulated splicing program is important for maintaining the epithelial phenotype. Over-expressing Esrp1-V1, a variant isoform of Esrp1, perturbs the Esrp1 regulated splicing program and leads to changes in gene expression and morphology that are associated with EMT.

We used Esrp1-V1 to test our hypothesis that if a gene promoted EMT it would inhibit tumor progression. Tumors formed by 4T1-Esrp1-V1 cells were significantly smaller than controls (*p* = 0.033, two-tailed student *t* test) (Figure 5G). At four weeks post tumor implantation, the average size of 4T1-Esrp1-V1 tumors was only 37% of the size of control tumors (Figure 5G). Lung metastatic tumor lesions were also significantly reduced in mice bearing 4T1-Esrp1-V1 tumors (11 lung metastatic tumor lesions in 4T1-control versus 1 lung metastatic tumor lesion in 4T1-Esrp1-V1) (Figure 5H). These data support our hypothesis that a gene promoting EMT would inhibit tumor growth and metastasis.

### Over-expression of *Grhl2* correlates with poor relapse-free survival and increased risk of metastasis in breast cancer patients

**Figure 6:**
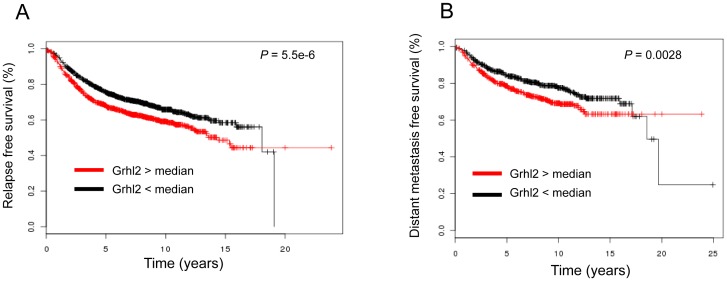
Over-expression of *Grhl2* correlates with shorter relapse-free survival interval and increased risk of metastasis in breast cancer patients. These analyses were performed using an online tool [17]. (A) Kaplan-Meier curve representation of probability of relapse-free survival in 2,898 patients with breast cancers according to expression levels of Grhl2. *P* values were calculated based on logrank tests. (B) Kaplan-Meier curve representation of probability of distant metastasis free survival in 1,354 patients with breast cancers according to expression levels of Grhl2. *P* values were calculated based on logrank tests.

Data from mouse models show that over-expression of Grhl2 promotes epithelial gene expression and promotes tumor progression. Examination of expressions of *Grhl2* and *E-cadherin* in human breast cancers reveals that these epithelial genes are not only expressed in primary tumors (Figure S6A, S6B) but also a majority of metastatic tumors (Figure S6C). To evaluate the clinical relevance of Grhl2 in breast cancer patients, we used a large public microarray database [17]. Clinical data revealed that higher expression levels of *Grhl2* were significantly correlated with a shorter relapse-free survival interval (2898 patients, *p* = 5.5E-6) and increased risk of metastasis (1354 patients, *p* = 0.0028) (Figure 6A 6B). Consistently, higher expression levels of other epithelial markers were significantly associated with poor relapse free survival and poor distant metastasis free survival, including *E-cadherin*, *Cldn3*, *Esrp1*, and *Epcam* (Figure S6D S6E). In addition, the 4T1 series, including 4T1 and 4TO7, are near-isogenic mouse mammary tumor cell lines. 4TO7 cells are able to disseminate to lung but have a very low efficiency to form metastatic lesions. Only 4T1 cells are able to complete the whole metastatic process and successfully establish metastases [5]. We found Grhl2 was only expressed in highly metastatic 4T1 cells but not weakly metastatic 4TO7 cells (Figure S6F S6G). Above all, these data indicate that *Grhl2* is associated with an unfavorable outcome in breast cancer patients.

## Discussion

### Grhl2 determines the epithelial phenotype of breast cancers

Using the 4T1 tumor model, we found *Grhl2* to be significantly down-regulated in disseminated cancer cells that had undergone EMT. Extensive bioinformatics analyses of large breast cancer microarray datasets revealed a remarkable level of correlation between the expression of *Grhl2* and *E-cadherin*. Knockdown of *Grhl2* in the human mammary epithelial cell line MCF10A led to down-regulation of *E-cadherin* and induction of EMT. Multiple functional assays confirm that Grhl2 plays a dominant role in maintaining the epithelial phenotype, and changing Grhl2 levels in cancer cells leads to EMT/MET induction. Collectively, these data indicate that Grhl2 is the essential transcription factor that determines the epithelial phenotype of breast cancers.

Our studies reveal that Grhl2 regulates a broad range of epithelial genes, including epithelial specific splicing factor (*Esrp1*), cell adhesion and tight junction (*E-cadherin*, *Tjp2*, *Cldn4* and *Cldn7*), Wnt ligands (*Wnt7A*, *Wnt7B*), and possibly microRNA (miR-200s). A previous study has reported that in Grhl2 mutant embryos, Esrp1 was significantly down-regulated [10] suggesting that the relationship between Grhl2 and Esrp1 was probably conserved across all types of epithelial cells. Recent studies suggest Wnt7A may play an important role in regulating epithelial cell differentiation in mice. Expression of Wnt7A is tightly co-regulated with epithelial genes during genital tubercle development [18], and in uterine Wnt7A is specifically expressed in epithelial cells [19], [20].

Members of the miR-200 family are epithelial specific microRNAs and play a critical role in EMT by targeting Zeb1 and Zeb2 [21], [22]. One study reported that expression of miR-200s is negatively regulated by Zeb1 and Zeb2 through the conserved E-box sites located within their promoter regions [23]. However, the transcription factor that drives the expressions of miR-200s in epithelial cells remains unidentified. In our study, we observed a strong correlation between the expression of Grhl2 and miR-200s. In Grhl2 knockdown MCF10A cells, the transcription level of miR-200c∼141 cluster was decreased. In 4T1 cells that had undergone EMT, Grhl2 was not expressed and transcription levels of miR-200s were very low. When Grhl2 was over-expressed, expression levels of the miR-200 family were up-regulated. Our data suggest that Grhl2 could be the transcription factor that drives the expression of miR-200 in breast cancer cells.

Several transcription factors, e.g. Gata3 and Klf4, have been identified that could induce E-cadherin expression through binding to E-cadherin promoter in human breast cancer cells [24], [25]. Gata3 plays a key role in determining luminal lineage cell differentiation [26], [27]. Microarray data clearly showed that *Gata3* was specifically expressed in luminal subtype cancer cells (Figure S2C). Basal A subtype tumors expressed very low level of *Gata3*, but high levels of *E-cadherin* and *Grhl2* (Figure S2C), indicating *Gata3* was not the essential transcription factor that controls E-cadherin expression in human breast cancers. Klf4 also has been reported to regulate E-cadherin expression via binding to the E-cadherin promoter region [25]. However, examination of *Klf4* expression in a panel of human breast cancer cells showed no correlation between *Klf4* and *E-cadherin* (Pearson correlation coefficient *r* = −0.05879, dataset GSE12777, 51 human breast cancer cells), indicating that although Klf4 was able to restore E-cadherin in mesenchymal breast cancer cells [25], it is not the essential transcription factor that determines the epithelial phenotype of breast cancer cells. Klf17 has been identified as a negative regulator of EMT by targeting Id1 [28]. Knockdown of *Klf17* led to EMT and promoted tumor metastasis [28]. Examination of *Klf17* expression in a panel of human breast cancer cells revealed no correlation between *Klf17* and *E-cadherin* (Pearson correlation coefficient *r* = −0.1407, dataset GSE12777). There was no correlation between *Id1* and *E-cadherin* either (Pearson correlation coefficient *r* = −0.033). Our data showed that Grhl2 was the only transcription factor that had such an extraordinary correlation with E-cadherin expression, and multiple functional assays confirmed that changes of epithelial gene expressions were strongly associated with Grhl2 expression during tumor progression. Our study indicates that Grhl2 is the essential transcription factor that determines the epithelial phenotypes of breast cancers. Our data also indicate that down-regulation of Grhl2 expression during tumor progression contributes to loss of epithelial phenotype and epithelial-to-mesenchymal transition.

### Grhl2 plays a critical role in epithelial-to-mesenchymal transition

During EMT, epithelial genes are down-regulated along with Grhl2, which is the essential transcription factor that controls epithelial gene expression. Down-regulation of Grhl2 could be a necessary step during EMT. In our studies, Grhl2 is significantly down-regulated in early disseminated 4T1 cells that have undergone EMT. Over-expression of either Wnt7A2 or Esrp1-V1 in 4T1 cells caused the cells to undergo EMT, and led to down-regulation of Grhl2. MCF10A cells were induced to undergo EMT by TGFβ (Figure S7A). During TGFβ induced EMT, Snai1 and Zeb1 are induced, but Grhl2 is down-regulated (Figure S7B). Further examination of publicly available microarray datasets also gave the same conclusion, which is that Grhl2 is always down-regulated in cells that have undergone EMT. Human mammary epithelial cells were induced to undergo EMT by Zeb1, Twist1 or Zeb2 combined with TGFβ [29], and Grhl2 was significantly down-regulated by these EMT inducers (Figure S7C). In a sub-population of spontaneously arising mesenchymal cells from immortalized human mammary epithelial cells (HMLE), expression level of Grhl2 is very low (GSE28681) [30]. In a sub-population of CD44^+^CD24^−^ cells derived from MCF10A cells, expression levels of Grhl2 is very low compared to CD44^−^CD24^+^ sub-population (GSE15192) [31]. Breast cancer cells derived from MMTV-NEU mouse were induced to undergo EMT *in vivo* after implanted into syngeneic mice, and Grhl2 is dramatically down-regulated in the cells that have undergone EMT (GSE13259) [32]. Snai1, Zeb1 and Zeb2 suppress gene expression by binding to E-box sites located within the promoter region of their targeted genes [23], [33], [34]. Two conserved E-box sites are located within the proximal promoter region of *Grhl2* (Figure 1E), suggesting that *Grhl2* may be suppressed directly by Snai1, Zeb1 or Zeb2. Down-regulation of *Grhl2* eventually leads to down-regulation of Grhl2 regulated genes, including E-cadherin, Epcam and Claudin4 [9], which contribute to inhibiting epithelial gene expression during the EMT process. Collectively, these data indicate that no matter what signaling pathway induces breast cancer cells to undergo EMT, Grhl2 is always down-regulated.

Grhl2 regulates a broad range of epithelial specific genes, and some of these genes can feedback to regulate the expression of Grhl2 and epithelial genes. Wnt7A is a Grhl2 regulated gene in 4T1 cells. Over-expression of Wnt7A leads to up-regulation of Grhl2 and E-cadherin. Microarray data reveal that genes that are up-regulated by Wnt7A with the highest fold changes are the same genes that are up-regulated by Grhl2, suggesting that Wnt7A mediated signaling could induce epithelial gene expression through induction of Grhl2. Interestingly, over-expressing a truncated Wnt7A isoform, Wnt7A2, which could potentially compete with the full length Wnt7A for binding to Wnt receptor or some other mechanism, leads to down-regulation of Grhl2 and E-cadherin. These data also suggest that Grhl2 and Wnt7A may form a positive feedback loop, and this signaling loop is important for maintaining epithelial status. Esrp1 is another Grhl2 regulated gene. Recent studies have identified Esrp1 as an epithelial specific splicing factor that controls the epithelial specific splicing of its targeted genes [16], [35]. Knockdown of Esrp1 in human mammary epithelial cells leads to epithelial cells undergoing some of the changes in gene expression and morphology that are associated with EMT [35], which suggests that the Esrp1 regulated splicing program is critical for maintaining the epithelial phenotype. In our study, we identified Esrp1 as a Grhl2 regulated gene. We confirmed that splicing of four Esrp1 regulated genes, including CTNND1 (P120 catenin), CD44, MAP3K7, and Enah, switched from the epithelial isoform to the mesenchymal isoform in 4T1 cells that had undergone EMT. Furthermore, we found that over-expressing a variant isoform of Esrp1, Esrp1-V1, perturbed the Esrp1 regulated splicing program and led to down-regulation of E-cadherin and Grhl2. Collectively, these data suggest that Grhl2 regulated genes also can feedback to regulate the expression of Grhl2 and epithelial phenotype.

### Grhl2 promotes tumor progression

Our results showed that over-expression of *Grhl2* significantly promoted tumor growth and metastasis. Further testing of several Grhl2 regulated genes, which either promoted EMT or inhibited EMT, always gave the same results, that the tumor growth and metastasis were linked to epithelial phenotype but not mesenchymal phenotype. In addition, we found Grhl2 was only expressed in highly metastatic 4T1 cells, but not weakly metastatic 4TO7 cells. Large clinical data also revealed that high levels of Grhl2 expression were significantly associated with poor relapse free survival and increased risk of metastasis in breast cancer patients.

It has been widely accepted that EMT plays a critical role during tumor metastasis [2]. Recently, several studies have reported conflicting roles of EMT in tumor growth and metastasis [36]–[38]. One study reported that over-expression of the miR-200s family was associated with increased risk of metastasis in breast cancer, and promoted metastatic colonization in mice models [36]. Clinical observations revealed that metastatic tumors were epithelial type [2], [13], [39]. Examination of large microarray datasets revealed that Grhl2 was not only expressed in primary tumors but also metastasis tumors. Tumor metastasis is a complex multistep process [40]. So far, very little is known about how the early disseminated cancer cells develop into metastasis tumors. Some researchers suggested that these early disseminated cancer cells might undergo mesenchymal-to-epithelial transition (MET) [2], which is based on observations that metastatic tumors were epithelial type. Given the essential role of Grhl2 in determining the epithelial phenotype, Grhl2 could play a pivotal role at the final step of tumor metastasis, and this possibility will require further studies.

## Materials and Methods

### Microarray hybridization and data analysis

RNAs isolated from 4T1-control cells recovered from primary tumors and lungs, 4T1-Grhl2, 4T1-Wnt7A1 and 4T1-Wnt7A2 cells recovered from lungs were processed for microarray hybridization using Affymetrix Murine GeneST Arrays according to the manufacturer's instructions (Affymetrix). Raw data were preprocessed by the robust multi-array analysis method as described previously [6].

Software MeV4.6 was used for microarray data analysis [41]. Dataset GSE12777 [14] and GSE16795 [15] were downloaded from the *Gene Expression Omnibus* (GEO) repository. According to the molecular definition of epithelial and mesenchymal breast cells [1], [12], [13], the following genes were set as epithelial markers: *Cdh1*, *Cldn3*, *Cldn4*, *Cldn7*, *Tjp2*, *Jup* and *CD24*; and these genes were set as markers for mesenchymal cells: *Cdh2*, *Vim* and *Zeb1*. Breast cancer cells were separated into an epithelial group and a mesenchymal group by Hierarchical clustering (HCL) analysis based on the transcription levels of epithelial markers and mesenchymal markers. A two-class Significance Analysis of Microarrays (SAM) was used to identify genes differentially expressed between the epithelial group and mesenchymal group (false discovery rate <5.00%). To search for epithelial-specific genes that co-regulated with E-cadherin in human breast cancer cells, all probes representing 54,675 transcripts were analyzed for differential expression in epithelial versus mesenchymal cells and Pearson correlation coefficient *r* was calculated between each transcript and E-cadherin across all microarrays. PAM305 breast cancer gene classifier [11] was used to identify molecular subtype.

The details of other methods used for this study, including cell culture, construction of lentiviral vectors, western blot analysis, RT-PCR and Real-time PCR, dual-luciferase assay, immunohistology staining, *in vivo* tumor growth and assays for metastasis have been published previously [6] and are described in Methods S1.

## Supporting Information

Figure S1 **Grh12 is down-regulated in 4T1 cancer cells recovered from lung that have undergone EMT.** (A) Schematic representation of recovering 4T1 cancer cells from primary tumors and lungs. 4T1 cells were subcutaneously injected in the mammary fat pad of BALB/c mice. At different time points after tumor implantation, mice were sacrificed and cancer cells were recovered from primary tumors and lungs. (B) Expression of E-cadherin and β-catenin in 4T1 cells recovered from primary tumors and lungs were examined by western blotting. Results represent one of four independent experiments (n = 18). (C) Relative levels of Grhl2 in 4T1 cells recovered from primary tumors and lungs were measured by quantitative realtime PCR. Error bars represent the mean ± SEM of duplicate experiments (n = 18). (D) Expression of Grhl2 in 4T1 cells recovered from primary tumors and lungs were examined by RT-PCT.
(PDF)

Figure S2 (A) All probes representing 54,675 transcripts on Dataset GSE12777 were analyzed for differential gene expression between epithelial and mesenchymal cells and Pearson correlation coefficient r was calculated between each transcript and vimentin across all microarrays. Some of the transcripts that were highly expressed in epithelial cells and negatively co-regulated with vimentin are labeled in Red, and some of the transcripts that were highly expressed in mesenchymal cells and co-regulated with vimentin are labeled in Blue. (B) Microarray-based screening for epithelial-specific transcription factors co-regulated with E-cadherin in human breast cancer cells (Dataset GSE12777). Probes representing about 3,000 transcripts that were annotated as transcription factor, DNA binding protein, or RNA binding protein, were analyzed for differential expression between epithelial and mesenchymal cells and Pearson correlation coefficient r was calculated between each transcript and E-cadherin. (C) Molecular subtypes in dataset GSE12777 were identified by PAM305 breast cancer subtype gene classifier [6]. The results show that these cancer cells can be separated into three groups, a luminal group expressing luminal and epithelial markers, a basal A group expressing myoepithelial and epithelial markers, and a basal B group expressing mesenchymal markers. Grhl2 is expressed in both luminal group and basal A group. (D) Hierarchical clustering of 39 human breast tumor cells in Dataset GSE16795 based on expression levels of epithelial and mesenchymal markers. Grhl2 is expressed exclusively in epithelial cells (P = 3.91754E-08, two-tailed students t tests).
(PDF)

Figure S3 **Stable expression of Grhl2 in 4T1 cells leads to up-regulation of primary microRNA of miR-200b∼200a∼429 cluster and a slight down-regulation of Zeb2.** Grhl2 was introduced into 4T1 cells by lentiviral infection. One week post virus transfection GFP+ cells were sorted. (A) Gene expression in 4T1-control and 4T1-Grhl2 cells was analyzed by quantitative realtime PCR. (B) Epcam expression was examined by staining with APC-labeled anti-Epcam antibody.
(PDF)

Figure S4 **Schematic representation of gene structure of two isoforms of mouse Wnt7A.** (A)Two cDNA clones differing in about 155 base pairs were obtained by using specific primers to amplify the full length open reading frame of mouse Wnt7A from a cDNA pool derived from 4T1 cells. Sequencing results showed that the longer cDNA shared 100% sequence identity with the reference sequence of mouse Wnt7A (NM_009527.3), which encoded a protein 349 amino acids in length. This isoform is designated as Wnt7A isoform 1, or Wnt7A1. The shorter cDNA lacks 155 base pairs compared to the reference sequence of mouse Wnt7A, which is due to using an alternative 5′ splice site in the exon 3. This results in introducing an in-frame stop codon immediately after the alternative splicing site. This isoform encodes a truncated Wnt7A protein 148 amino acids in length. This isoform is designated as Wnt7A isoform 2 or Wnt7A2. (B) Schematics represent two alternative splicing sites that generate Wnt7A1 and Wnt7A2. Mouse Wnt7A exon3 is labeled in red and green colors, and adjacent introns were labeled in black colors. The sequence excluded in Wnt7A2 is labeled in green color. Two splicing sites are underline labeled. These two splicing sites share a sequence similarity. (C) Wnt7A1 and Wnt7A2 were introduced into 4T1 cells by lentiviral infection. Gene expressions in 4T1-control, 4T1-Wnt7A1 and 4T1-Wnt7A2 cells were analyzed by quantitative realtime PCR.
(PDF)

Figure S5 (A) Esrp1 was down-regulated in 4T1 cells recovered from lung, which corresponded with its targeted gene expression switching from the epithelial isoform to the mesenchymal isoform. Left schematics depict alternative splicing events and small arrows representing primers used for RT-PCR. (B) Schematics represent an alternative splicing event in Esrp1-V1. Esrp1 Exon12 sequence is underlined and the adjacent intron sequence is labeled in black. The sequence included in Esrp1-V1 is labeled in red, and the sequence excluded in Esrp1- V1 is labeled in green. The 5′ splicing site used by Esrp1 and alternative splicing site used by Esrp1-V1 were highlighted by purple bars beneath the sequence. These two splicing sites share a remarkably high sequence identity, and in addition, are conserved across human and mouse. (C) Searching the EST database retrieved many EST sequences that shared the exact same splicing sites with Esrp1-V1, including many EST sequences from mouse and human samples, indicating using this splicing site is very common. Accession numbers of each sequence were placed in front each of sequence. (D) Stable expression of Esrp1-V1 led to switching of Esrp1 regulated genes from an epithelial isoform to a mesenchymal isoform. 4T1 cells recovered from lung that had undergone EMT were used as the control to show the mesenchymal isoform.
(PDF)

Figure S6 (A) Examination of expression levels of Grhl2 and other EMT markers in breast cancer primary tumors. Dataset GSE12276 contains microarray data of 204 primary breast tumors. Heatmap showing expression levels of epithelial markers (Cdh1, Grhl2, Epcam, Esrp1, Cldn3, Cldn4, Cldn7, and JUP), mesenchymal markers (Zeb1, Zeb2 and Cdh2), myoepithelial cell markers and luminal cell markers in primary breast tumors. The heatmap clearly shows that Grhl2 and other epithelial markers are expressed in almost all breast cancer primary tumors. And similar results were obtained from by analyzing another dataset GSE2034 (B). (C) Examination of expression levels of Grhl2 and other EMT markers in breast cancer metastatic tumors. Dataset GSE14017 contains microarray data of 29 breast cancer metastatic tumors from different organs (4 lung metastases, 15 brain metastases, and 10 bone metastases). The heatmap shows that expression patterns of these genes in metastasis tumors are similar to primary tumors. Grhl2 and other epithelial markers are highly expressed in majority of metastasis tumors. (D) Kaplan-Meier curve representation of probability of relapse-free survival in 2,898 patients with breast cancers according to expression levels of epithelial and mesenchymal markers. P values were calculated based on logrank tests. These analyses were performed by an online tool [7]. (E) Kaplan-Meier curve representation of probability of distant metastasis free survival in 1,354 patients with breast cancers according to expression levels of epithelial markers. P values were calculated based on logrank tests. These analyses were performed by an online tool [7]. (F) Expression of Grhl2 mRNA in 4T1 cells recovered from primary tumors and lungs, and 4TO7 cells were examined by RT-PCR. Results represent one of five independent experiments. (G) Relative expression levels of Grhl2 as well as other EMT markers in 4T1 and 4TO7 cells were measured by quantitative realtime PCR.
(PDF)

Figure S7 (A) Grhl2 is down-regulated during TGFβ induced EMT. MCF10A cells were induced to undergo EMT by TGFβ. Four days post TGFβ (5 ng/ml) treatment, MCF10A cells were transformed from cobble-stone like epithelial morphology to spindle-like mesenchymal morphology (upper penal), with disruption of cell-cell border E-cadherin staining (middle penal) and increasing vimentin staining (bottom penal). The images are representative one of five independent experiments. (B) Relative expression levels of Grhl2 mRNA in MCF10A cells treated with TGFβ or untreated were measured by quantitative realtime PCR. Error bars represent mean ± SEM of three experiments. (C) We analyzed publicly available microarray datasets to see if Grhl2 was downregulated by EMT inducers in human mammary epithelial cells (HMEC). These data, which are up-loaded by stéphane ansieau, include microarray data of immortalized human mammary epithelial cells (HMEC-hTert) or HMEC-hTert cells transduced with HRasG12V (HMEC-hTert-Ras) over-expressing EMT inducer Zeb1, Zeb2 or Twist1 [8]. These data reveal that down-regulation of E-cadherin (Cdh1) by Zeb1, Twsit1, or Zeb2 combined with TGFβ, also cause in down-regulation of Grhl2 expression. And similar expression changes are also observed for Esrp1. These data indicate that Grhl2 is down-regulated during EMT.
(PDF)

Methods S1 Supplementary Material and Methods.
(PDF)

Table S1 Genes significantly down- or up- regulated in disseminated 4T1 cells that have undergone EMT. Expression level was Log2 transformed.
(PDF)

Table S2 Comparison of gene expression profiles among 4T1-control cells recovered from primary tumors and lungs, 4T1-Grhl2, 4T1-Wnt7A1 and 4T1-Wnt7A2 cells recovered from lungs. Transcription level was Log2 transformed.
(PDF)

Table S3 Primers used for Realtime PCR and RT-PCR.
(PDF)

Table S4 Primers used for constructing lentiviral and retroviral vectors.
(PDF)
