# Correction: Grhl2 Determines the Epithelial Phenotype of Breast Cancers and Promotes Tumor Progression

**DOI:** 10.1371/annotation/aa633603-370b-4613-bf35-2e5f999cff38

**Published:** 2013-04-08

**Authors:** Xiaoyu Xiang, ZhongBin Deng, Xiaoying Zhuang, Songwen Ju, Jingyao Mu, Hong Jiang, Lifeng Zhang, Jun Yan, Donald Miller, Huang-Ge Zhang

In our article we report that Grhl2 plays an essential role in the determination of epithelial phenotype of breast cancers, epithelial-to-mesenchymal transition (EMT) and tumor progression. A previous publication by Cieply et al. had reported a role for GRHL2 in the suppression of oncogenic EMT in breast cancer cells and the authors regret not citing this article:

Cieply B, Riley Pt, Pifer PM, Widmeyer J, Addison JB, Ivanov AV, Denvir J, Frisch SM: Suppression of the epithelial-mesenchymal transition by Grainyhead-like-2. Cancer Res 2012, 72:2440-53.

At the time at which we were carrying out our experiments, one study had reported that Grhl2 regulated adhesion junction gene E-cadherin and the tight junction gene claudin 4 (Cldn4) in several types of epithelial cells (Werth et al. The transcription factor grainyhead-like 2 regulates the molecular composition of the epithelial apical junctional complex. Development 2010, 137:3835-45.). However, no study had addressed its role in terms of growth of breast cancer cells and breast cancer metastasis.

The work by Cieply et al. focused on the role of Grhl2 in suppressing epithelial-to-mesenchymal transition, they reported that Grhl2 acted in part by suppression of Zeb1 expression via direct repression of Zeb1 promoter. They concluded that their findings defined a major role of Grhl2 in the suppression of oncogenic EMT in breast cancer cells.

Both our data and those reported by Cieply et al. support that Grhl2 suppresses EMT. Grhl2 is expressed in breast cancer cells in epithelial status and is down-regulated when tumor cells are induced to undergo EMT by signaling pathways such as TGFβ. Knockdown of Grhl2 in human mammary epithelial cells leads to a loss of epithelial morphology, epithelial markers and induction of EMT. Grhl2 directly or indirectly regulates a broad range of epithelial genes. All these data indicate a physiological role of Grhl2 in regulating epithelial gene expression and epithelial phenotype in breast cancer cells, as reported in our article.

Our study included the role of Grhl2 in tumor progression in animal models, which was not tested by Cieply et al. We also tested two of the genes regulated by Grhl2, analyzed publically available clinical data to evaluate the correlation between the expression of Grhl2 and outcome in breast cancer patients and checked its expression levels in metastasis tumors from breast cancer patients. 

