# Supplementary material for: Grhl2 Determines the Epithelial Phenotype of Breast Cancers and Promotes Tumor Progression
Source: PLoS One. 2012 Dec 17;7(12):e50781. doi: 10.1371/journal.pone.0050781 (PMC3524252; doi:10.1371/journal.pone.0050781)
Supplement: Figure S1 — Grh12 is down-regulated in 4T1 cancer cells recovered from lung that have undergone EMT. (A) Schematic representation of recovering 4T1 cancer cells from primary tumors and lungs. 4T1 cells were subcutaneously injected in the mammary fat pad of BALB/c mice. At different time points after tumor implantation, mice were sacrificed and cancer cells were recovered from primary tumors and lungs. (B) Expression of E-cadherin and β-catenin in 4T1 cells recovered from primary tumors and lungs were examined by western blotting. Results represent one of four independent experiments (n = 18). (C) Relative levels of Grhl2 in 4T1 cells recovered from primary tumors and lungs were measured by quantitative realtime PCR. Error bars represent the mean ± SEM of duplicate experiments (n = 18). (D) Expression of Grhl2 in 4T1 cells recovered from primary tumors and lungs were examined by RT-PCT. (PDF) [file pone.0050781.s001.pdf]

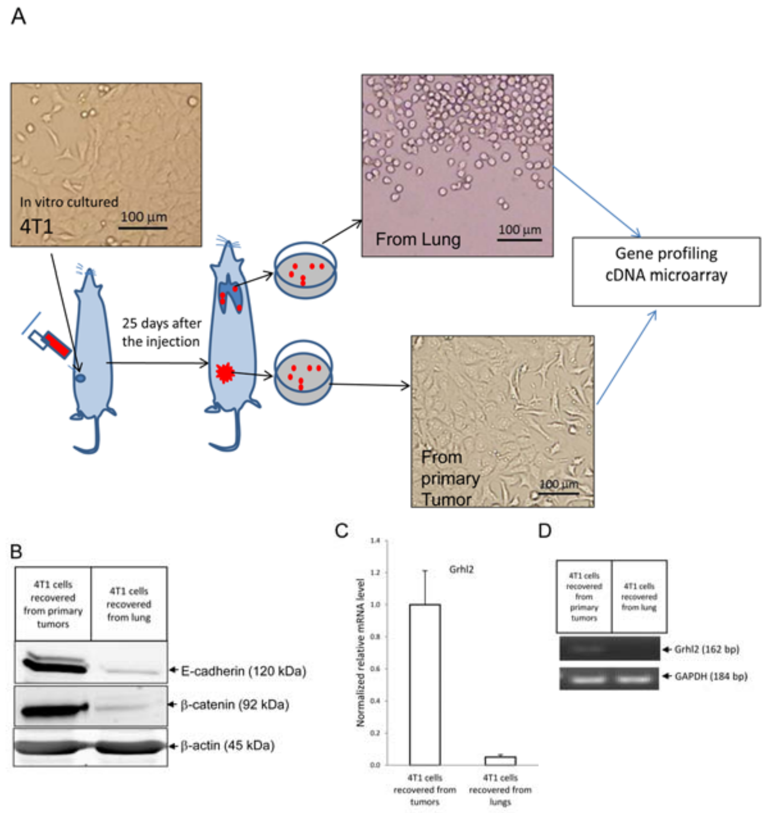

Figure S1 Grh12 is down-regulated in 4T1 cancer cells recovered from lung that have undergone EMT. (A) Schematic representation of recovering 4T1 cancer cells from primary tumors and lungs. 4T1 cells were subcutaneously injected in the mammary fat pad of BALB/c mice. At different time points after tumor implantation, mice were sacrificed and cancer cells were recovered from primary tumors and lungs.

(B) Expression of E-cadherin and □-catenin in 4T1 cells recovered from primary tumors and lungs were examined by western blotting. Results represent one of four independent experiments (n=18). (C) Relative levels of Grhl2 in 4T1 cells recovered from primary tumors and lungs were measured by quantitative realtime PCR. Error bars represent the mean ± SEM of duplicate experiments (n=18).

(D) Expression of Grhl2 in 4T1 cells recovered from primary tumors and lungs were examined by RT-PCT.
