# Supplementary material for: Grhl2 Determines the Epithelial Phenotype of Breast Cancers and Promotes Tumor Progression
Source: PLoS One. 2012 Dec 17;7(12):e50781. doi: 10.1371/journal.pone.0050781 (PMC3524252; doi:10.1371/journal.pone.0050781)
Supplement: Figure S3 — Stable expression of Grhl2 in 4T1 cells leads to up-regulation of primary microRNA of miR-200b∼200a∼429 cluster and a slight down-regulation of Zeb2. Grhl2 was introduced into 4T1 cells by lentiviral infection. One week post virus transfection GFP+ cells were sorted. (A) Gene expression in 4T1-control and 4T1-Grhl2 cells was analyzed by quantitative realtime PCR. (B) Epcam expression was examined by staining with APC-labeled anti-Epcam antibody. (PDF) [file pone.0050781.s003.pdf]

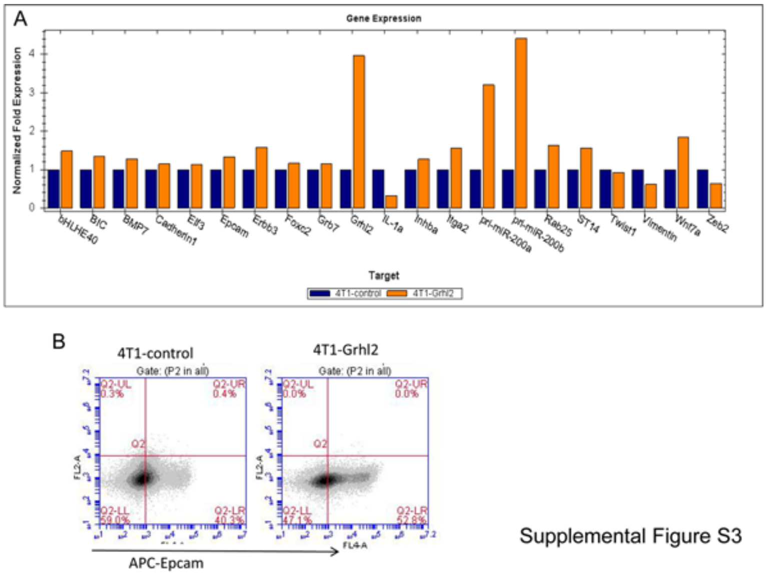

Supplemental Figure S3

Figure S3 Stable expression of *Grhl2* in 4T1 cells leads to up-regulation of primary microRNA of miR-200b~200a~429 cluster and a slight down-regulation of Zeb2. Grhl2 was introduced into 4T1 cells by lentiviral infection. One week post virus transfection GFP^+ cells were sorted. (A) Gene expression in 4T1-control and 4T1-Grhl2 cells was analyzed by quantitative realtime PCR. (B) Epcam expression was examined by staining with APC-labeled anti-Epcam antibody.
