# Supplementary material for: Grhl2 Determines the Epithelial Phenotype of Breast Cancers and Promotes Tumor Progression
Source: PLoS One. 2012 Dec 17;7(12):e50781. doi: 10.1371/journal.pone.0050781 (PMC3524252; doi:10.1371/journal.pone.0050781)
Supplement: Figure S5 — (A) Esrp1 was down-regulated in 4T1 cells recovered from lung, which corresponded with its targeted gene expression switching from the epithelial isoform to the mesenchymal isoform. Left schematics depict alternative splicing events and small arrows representing primers used for RT-PCR. (B) Schematics represent an alternative splicing event in Esrp1-V1. Esrp1 Exon12 sequence is underlined and the adjacent intron sequence is labeled in black. The sequence included in Esrp1-V1 is labeled in red, and the sequence excluded in Esrp1- V1 is labeled in green. The 5′ splicing site used by Esrp1 and alternative splicing site used by Esrp1-V1 were highlighted by purple bars beneath the sequence. These two splicing sites share a remarkably high sequence identity, and in addition, are conserved across human and mouse. (C) Searching the EST database retrieved many EST sequences that shared the exact same splicing sites with Esrp1-V1, including many EST sequences from mouse and human samples, indicating using this splicing site is very common. Accession numbers of each sequence were placed in front each of sequence. (D) Stable expression of Esrp1-V1 led to switching of Esrp1 regulated genes from an epithelial isoform to a mesenchymal isoform. 4T1 cells recovered from lung that had undergone EMT were used as the control to show the mesenchymal isoform. (PDF) [file pone.0050781.s005.pdf]

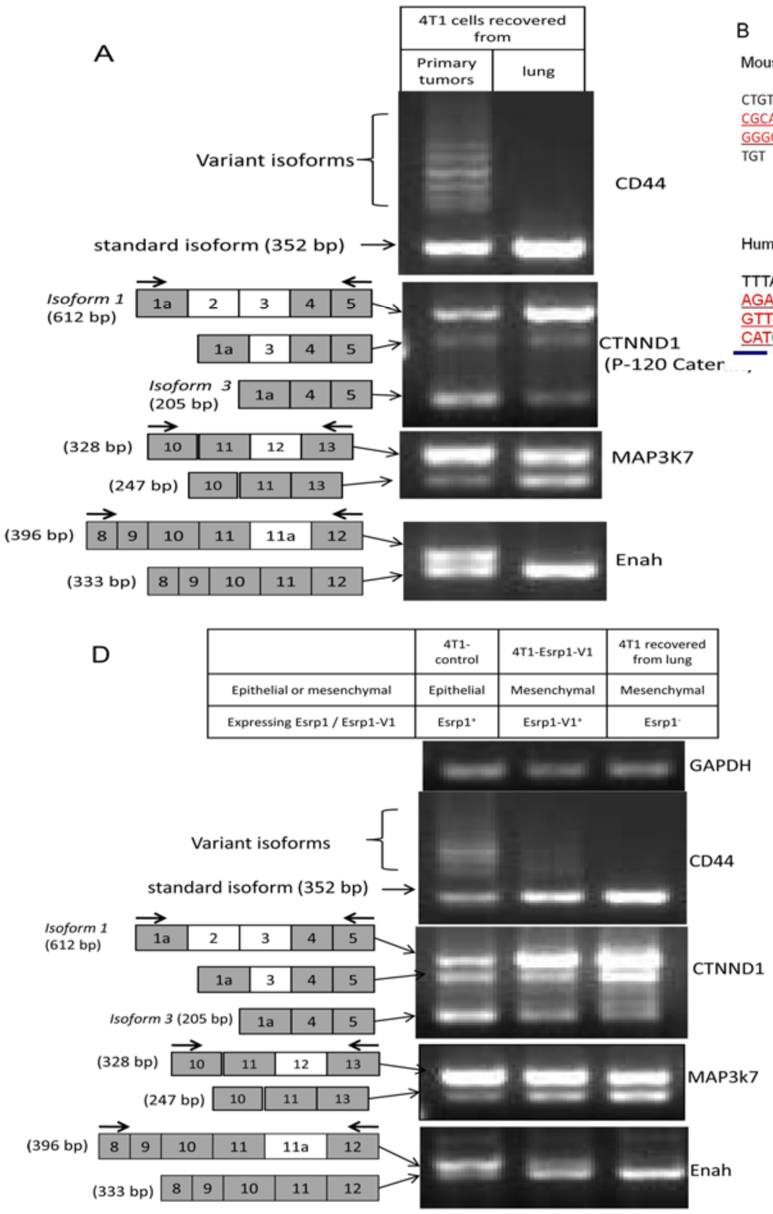

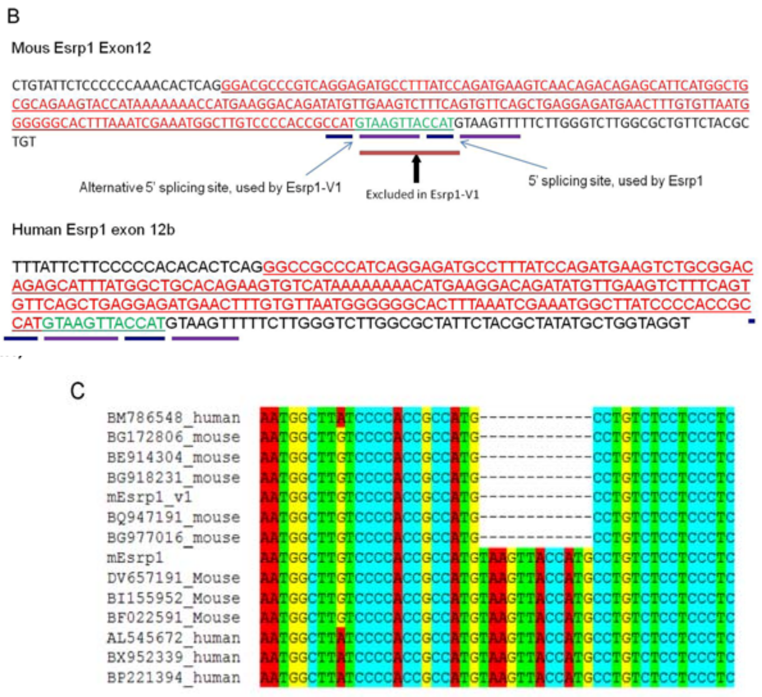

Figure S5 (A) *Esrp1* was down-regulated in 4T1 cells recovered from lung, which corresponded with its targeted gene expression switching from the epithelial isoform to the mesenchymal isoform. Left schematics depict alternative splicing events and small arrows representing primers used for RT-PCR. (B) Schematics represent an alternative splicing event in Esrp1-V1. Esrp1 Exon12 sequence is underlined and the adjacent intron sequence is labeled in black. The sequence included in Esrp1-V1 is labeled in red, and the sequence excluded in Esrp1-V1 is labeled in green. The 5' splicing site used by Esrp1 and alternative splicing site used by Esrp1-V1 were highlighted by purple bars beneath the sequence. These two splicing sites share a remarkably high sequence identity, and in addition, are conserved across human and mouse.

(C) Searching the EST database retrieved many EST sequences that shared the exact same splicing sites with Esrp1-V1, including many EST sequences from mouse and human samples, indicating using this splicing site is very common. Accession numbers of each sequence were placed in front each of sequence.

(D) Stable expression of Esrp1-V1 led to switching of Esrp1 regulated genes from an epithelial isoform to a mesenchymal isoform. 4T1 cells recovered from lung that had undergone EMT were used as the control to show the mesenchymal isoform.
