# Supplementary material for: Grhl2 Determines the Epithelial Phenotype of Breast Cancers and Promotes Tumor Progression
Source: PLoS One. 2012 Dec 17;7(12):e50781. doi: 10.1371/journal.pone.0050781 (PMC3524252; doi:10.1371/journal.pone.0050781)
Supplement: Figure S6 — (A) Examination of expression levels of Grhl2 and other EMT markers in breast cancer primary tumors. Dataset GSE12276 contains microarray data of 204 primary breast tumors. Heatmap showing expression levels of epithelial markers (Cdh1, Grhl2, Epcam, Esrp1, Cldn3, Cldn4, Cldn7, and JUP), mesenchymal markers (Zeb1, Zeb2 and Cdh2), myoepithelial cell markers and luminal cell markers in primary breast tumors. The heatmap clearly shows that Grhl2 and other epithelial markers are expressed in almost all breast cancer primary tumors. And similar results were obtained from by analyzing another dataset GSE2034 (B). (C) Examination of expression levels of Grhl2 and other EMT markers in breast cancer metastatic tumors. Dataset GSE14017 contains microarray data of 29 breast cancer metastatic tumors from different organs (4 lung metastases, 15 brain metastases, and 10 bone metastases). The heatmap shows that expression patterns of these genes in metastasis tumors are similar to primary tumors. Grhl2 and other epithelial markers are highly expressed in majority of metastasis tumors. (D) Kaplan-Meier curve representation of probability of relapse-free survival in 2,898 patients with breast cancers according to expression levels of epithelial and mesenchymal markers. P values were calculated based on logrank tests. These analyses were performed by an online tool [7]. (E) Kaplan-Meier curve representation of probability of distant metastasis free survival in 1,354 patients with breast cancers according to expression levels of epithelial markers. P values were calculated based on logrank tests. These analyses were performed by an online tool [7]. (F) Expression of Grhl2 mRNA in 4T1 cells recovered from primary tumors and lungs, and 4TO7 cells were examined by RT-PCR. Results represent one of five independent experiments. (G) Relative expression levels of Grhl2 as well as other EMT markers in 4T1 and 4TO7 cells were measured by quantitative realtime PCR. (PDF) [file pone.0050781.s006.pdf]

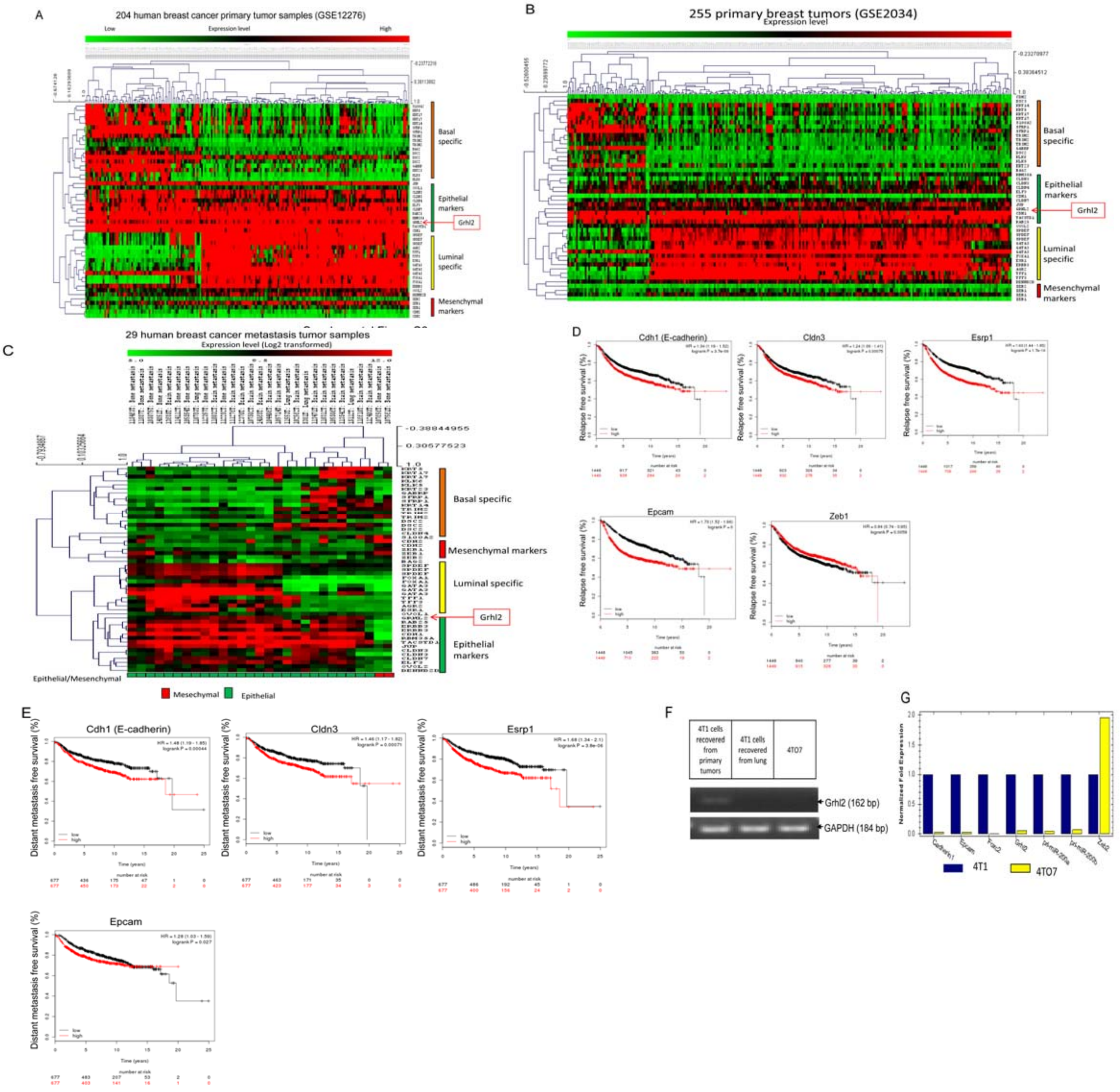

Figure S6 (A) Examination of expression levels of *Grhl2* and other EMT markers in breast cancer primary tumors. Dataset GSE12276 contains microarray data of 204 primary breast tumors. Heatmap showing expression levels of epithelial markers (Cdh1, Grhl2, Epcam, Esrp1, Cldn3, Cldn4, Cldn7, and JUP), mesenchymal markers (Zeb1, Zeb2 and Cdh2), myoepithelial cell markers and luminal cell markers in primary breast tumors. The heatmap clearly shows that *Grhl2* and other epithelial markers are expressed in almost all breast cancer primary tumors. And similar results were obtained from by analyzing another dataset GSE2034 (B).

(C) Examination of expression levels of *Grhl2* and other EMT markers in breast cancer metastatic tumors. Dataset GSE14017 contains microarray data of 29 breast cancer metastatic tumors from different organs (4 lung metastases, 15 brain metastases, and 10 bone metastases). The heatmap shows that expression patterns of these genes in metastasis tumors are similar to primary tumors. Grhl2 and other epithelial markers are highly expressed in majority of metastasis tumors.

(D) Kaplan-Meier curve representation of probability of relapse-free survival in 2,898 patients with breast cancers according to expression levels of epithelial and mesenchymal markers. *P* values were calculated based on logrank tests. These analyses were performed by an online tool [1].

(E) Kaplan-Meier curve representation of probability of distant metastasis free survival in 1,354 patients with breast cancers according to expression levels of epithelial markers. *P* values were calculated based on logrank tests. These analyses were performed by an online tool [1].

(F) Expression of Grhl2 mRNA in 4T1 cells recovered from primary tumors and lungs, and 4TO7 cells were examined by RT-PCR. Results represent one of five independent experiments.

(G) Relative expression levels of Grhl2 as well as other EMT markers in 4T1 and 4TO7 cells were measured by quantitative realtime PCR.
