# Supplementary material for: Grhl2 Determines the Epithelial Phenotype of Breast Cancers and Promotes Tumor Progression
Source: PLoS One. 2012 Dec 17;7(12):e50781. doi: 10.1371/journal.pone.0050781 (PMC3524252; doi:10.1371/journal.pone.0050781)
Supplement: Figure S7 — (A) Grhl2 is down-regulated during TGFβ induced EMT. MCF10A cells were induced to undergo EMT by TGFβ. Four days post TGFβ (5 ng/ml) treatment, MCF10A cells were transformed from cobble-stone like epithelial morphology to spindle-like mesenchymal morphology (upper penal), with disruption of cell-cell border E-cadherin staining (middle penal) and increasing vimentin staining (bottom penal). The images are representative one of five independent experiments. (B) Relative expression levels of Grhl2 mRNA in MCF10A cells treated with TGFβ or untreated were measured by quantitative realtime PCR. Error bars represent mean ± SEM of three experiments. (C) We analyzed publicly available microarray datasets to see if Grhl2 was downregulated by EMT inducers in human mammary epithelial cells (HMEC). These data, which are up-loaded by stéphane ansieau, include microarray data of immortalized human mammary epithelial cells (HMEC-hTert) or HMEC-hTert cells transduced with HRasG12V (HMEC-hTert-Ras) over-expressing EMT inducer Zeb1, Zeb2 or Twist1 [8]. These data reveal that down-regulation of E-cadherin (Cdh1) by Zeb1, Twsit1, or Zeb2 combined with TGFβ, also cause in down-regulation of Grhl2 expression. And similar expression changes are also observed for Esrp1. These data indicate that Grhl2 is down-regulated during EMT. (PDF) [file pone.0050781.s007.pdf]

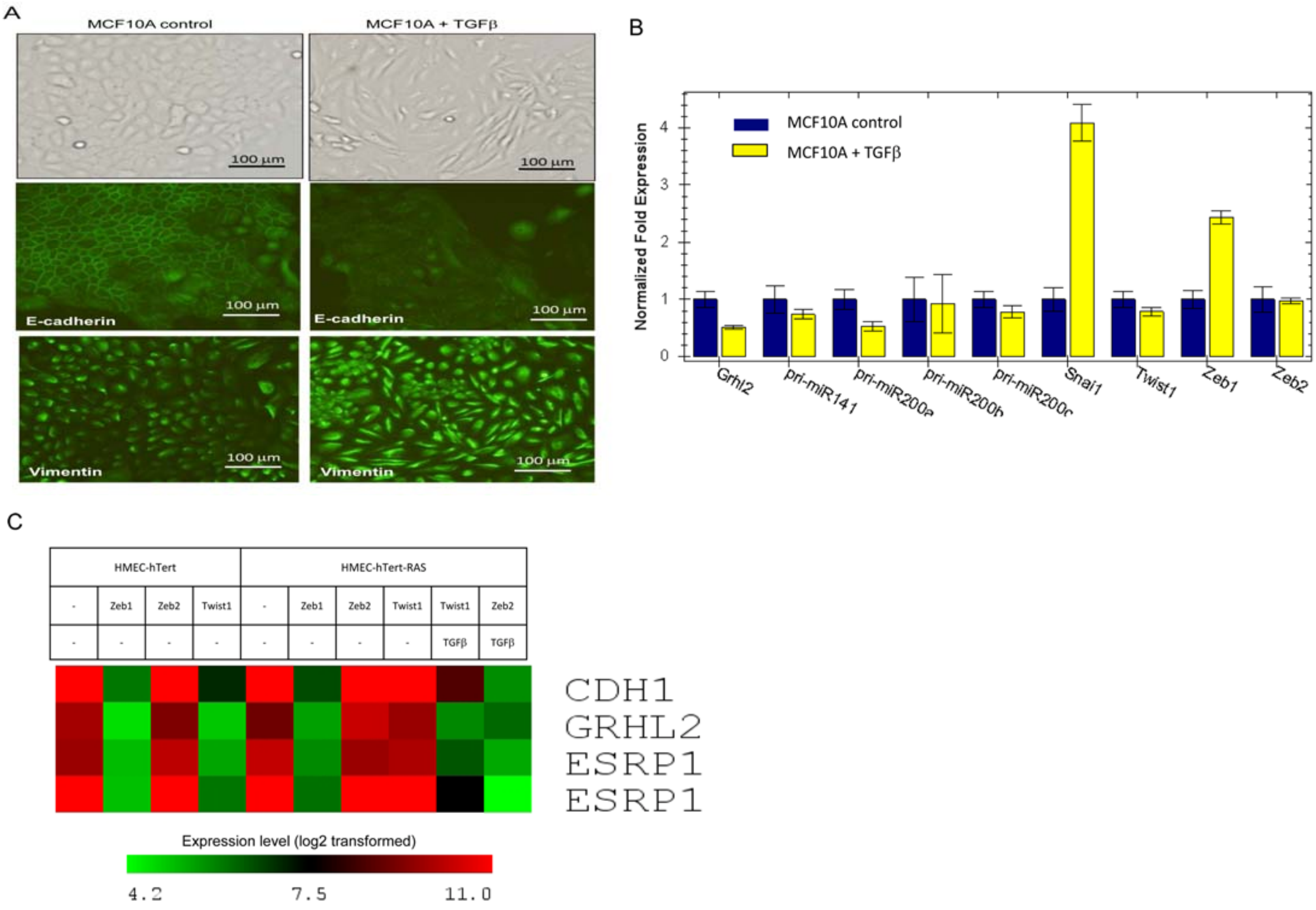

Figure S7 (A) *Grhl2* is down-regulated during TGFβ induced EMT. MCF10A cells were induced to undergo EMT by TGFβ. Four days post TGFβ (5ng/ml) treatment, MCF10A cells were transformed from cobble-stone like epithelial morphology to spindle-like mesenchymal morphology (upper penal), with disruption of cell-cell border E-cadherin staining (middle penal) and increasing vimentin staining (bottom penal). The images are representative one of five independent experiments.

(B) Relative expression levels of *Grhl2* mRNA in MCF10A cells treated with TGFβ or untreated were measured by quantitative realtime PCR. Error bars represent mean ± SEM of three experiments.

(C) We analyzed publicly available microarray datasets to see if Grhl2 was down-regulated by EMT inducers in human mammary epithelial cells (HMEC). These data, which are up-loaded by stéphane ansieau, include microarray data of immortalized human mammary epithelial cells (HMEC-hTert) or HMEC-hTert cells transduced with H-RasG12V (HMEC-hTert-Ras) over-expressing EMT inducer Zeb1, Zeb2 or Twist1 [2]. These data reveal that down-regulation of E-cadherin (Cdh1) by Zeb1, Twsit1, or Zeb2 combined with TGFβ, also cause in down-regulation of Grhl2 expression. And similar expression changes are also observed for Esrp1. These data indicate that Grhl2 is down-regulated during EMT.
