# Supplementary material for: Grhl2 Determines the Epithelial Phenotype of Breast Cancers and Promotes Tumor Progression
Source: PLoS One. 2012 Dec 17;7(12):e50781. doi: 10.1371/journal.pone.0050781 (PMC3524252; doi:10.1371/journal.pone.0050781)
Supplement: Methods S1 — Supplementary Material and Methods. (PDF) [file pone.0050781.s008.pdf]

Supplementary Material and Methods

Antibodies and Cell cultures

All antibodies, including mouse monoclonal anti-E-cadherin (BD), anti-vimentin (BD BioSciences), rabbit anti-Epcam monoclonal antibody (Epitomics Inc), rabbit anti-β catenin (from Cell Signaling), mouse anti-HA monoclonal antibody (Convance), monoclonal mouse anti-β actin (BioLegend), and APC labeled anti-Epcam antibody (Biolegend) were all diluted and used according to the manufacturer's instructions. 4T1, 293T, MDA-MB-231, MCF7, and Hs578T cell lines were purchased from ATCC and maintained in DMEM supplemented with 10% fetal bovine serum (FBS). T-47D cells were obtained from ATCC and maintained in RPMI-1640 medium supplemented with 10% FBS. MCF10A cells were maintained in DMEM/F12 media (Invitrogen), supplemented with 5% horse serum (Invitrogen), 20 ng/ml EGF, 0.5 mg/ml hydrocortisone (Sigma), 100 ng/ml cholera toxin (Sigma), 10 μg/ml insulin (Invitrogen), 100 U/mL penicillin, and 100 μg/mL streptomycin. All cells were grown in a humidified atmosphere of 5% CO_2 at 37°C.

RNA isolation, RT-PCR and Realtime PCR

RNAs were isolated with Trizol reagent (Invitrogen) according to the manufacturer's protocol without any modification. Five hundred ng of purified RNAs were used for cDNA synthesis using the SuperScript® III First-Strand Synthesis System (Invitrogen). For RT-PCR, 1 μl of cDNA product was used in a 20 μl PCR reaction containing 1 U Taq DNA polymerase (New England Biolab), 200 μM dNTP mixture, and primers. All

primers were purchased from Eurofins MWG Operon (Huntsville, AL). The sequence of primers is listed in a supplementary file (Supplementary table 3). Relative quantification of selected mRNAs was performed using a CFX96 Realtime System (BioRad) and SsoFast evagreen supermixture (Bio-Rad Laboratories, Hercules, CA) according to the manufacturer's instructions. Relative expression level was calculated after being normalized to GAPDH and β-actin expression.

Dual-luciferase assay

Plasmid pGL3-basic and pRL-TK were purchased from Promega. pGL2-E-cadherin promoter luciferase vector was a generous gift from Dr. Fearon [1]. The proximal promoter region of mouse *Grhl2* was cloned from mouse genomic DNA by *pfu* DNA polymerase with specific primers (mGrhl2 Promoter forward primer: acgcgtccaccacgctcatgtatgggcta, mGrhl2 Promoter reverse primer: aagcttgtttgatccaatgaactcgctcc) and then inserted into a pGL3-basic plasmid at the *Mlu*I and *Hind*III sites. The DNA sequence was verified by sequencing. Cells were seeded into 6-well plates one day before transfection. Cells were co-transfected with 1800 ng DNAs of pGL3-basic, pGL3-Grhl2-promoter, or pGL2-E-cadherin promoter, and 200 ng DNAs of pRL-TK (Renilla luciferase, Promega) with Fugene transfection reagent (Roche). Forty-eight hrs after transfection, luciferase activity was analyzed using a dual-luciferase assay kit (promega) according to the manufacturer’s protocol. Firefly luciferase activity was normalized to Renilla luciferase activity.

Retroviral and lentiviral constructs and virus package

Retroviral vector MDH1-PGK-GFP 2.0 was constructed by Chang-Zheng Chen [2], and was obtained from Addgene. This vector contains an H1 promoter that can be used to drive shRNA expression. shRNAs targeting human *Grhl2* or firefly luciferase were designed at Invitrogen webserver BLOCK-iT™ RNAi Designer. Oligo DNAs used for constructing shRNA vectors were listed in supplemental table 4. To package retrovirus capable of infecting human cells, 293T cells were seeded into 6-cm dishes one day before transfection. 293T cells were co-transfected with 2000 ng DNAs of MDH-PGK-GFP, 1600 ng DNAs of pCL, and 400 ng DNAs of pVsvg[3] with 10 µl Fugene HD transfection reagent (Roche). Twenty-four hours after transfection, cells received fresh media. Virus was harvested 48 hours after transfection. To infect human cells, the cells were seeded sparsely into 6 well plates one day before transfection. The following day the media were removed, cells were fed with 2 ml fresh media and 2 ml of freshly harvested virus with 2 ul of 10 mg/ml DEAE Dextran was added to the plate wells. GFP^+ cells were sorted one week after viral infection.

Lentiviral vector 2229 was a generous gift from Dr. John Kappes [3]. This vector contains a CMV promoter to drive expression of the genes of interest and IRES to select for GFP. Grhl2, Wnt7A, and Esrp1 were amplified from cDNA pools derived from 4T1 cells by pfu DNA polymerase. Primers used for cDNA cloning are listed in supplementary table 4. All cDNA clones were fully sequenced. To package lentivirus, 293T cells were seeded into 6-cm dishes one day before transfection. 293T cells were co-transfected with 2000 ng DNAs of the lentirival construct, 1600 ng DNAs of pDelta8.2, and 400 ng DNAs of pVsvg [3] (Dr. John Kappes) with 10 µl Fugene HD

transfection reagent (Roche). Cells were fed 4 ml of fresh media 24 hours post transfection. Virus was harvested 48 hours after transfection. To transfect cells, the cells were seeded sparsely into 6.0 cm dishes one day before infection. The following day the media were removed, and cells were fed with 2 ml fresh media and 2 ml of freshly harvested virus with 2 µl of 10 mg/ml DEAE Dextran was added. GFP^+ cells were sorted one week after viral infection.

In vivo tumor growth and metastatic assays

Female wild type BALB/c mice, 6 to 8 weeks old, were obtained from The Jackson Laboratory (Bar Harbor, ME). All procedures were approved by the University of Louisville Institutional Animal Care and Use Committee. Cancer cells ($1x10^5$) were injected in the mammary fat pads of mice in a volume of 100 µl of PBS. Tumors were measured every three days and volume was calculated using a method as described [4]. Mice were sacrificed 4 weeks post tumor transplantation and tumors and lungs were removed. Lungs were fixed in buffered fomalde-fresh solution (Fisher) and stained with hematoxylin and eosin (H&E) for counting metastatic nodules.

Recovering 4T1 tumor cells from primary tumors and lungs

Protocols used to recover 4T1 tumor cells from primary tumors and lungs have been described previously [5].

Immunofluorescent staining of E-cadherin and vimentin in cultured cells

Cells grown on coverslips were washed extensively in phosphate buffered saline (PBS) and fixed with 2% paraformaldehyde in PBS for 20 min. After additional washing, the cells were permeabilized with 0.2% Triton X-100 in PBS for 10 min. The cells were then washed and blocked with 5% BSA in PBS for 60 min. Cells were labeled with anti-E-cadherin antibody (BD Transduction) or anti-vimentin antibody (BD transduction) overnight at 4°C. The coverslips were washed extensively with PBS and the cells stained with Alexa 488-conjugated donkey anti-mouse antibody (Molecular Probes) for 60 min at 22°C. After further washing, the cover slips were mounted on slides and images were acquired using an Olympus (IX71) microscope equipped with DPController Software (Olympus).

Immunofluorescent staining of paraffin sections of fixed lung tissues

Lung tissues were dissected, fixed in buffered formalde-fresh solution (Fisher), embedded in paraffin, and cut into 4 μm sections. Paraffin was removed with two washes of xylene for five minutes each. Slides were then incubated in two washes of 100% ethanol for 10 minutes each, 95%, 85%, and 70% ethanol for 10 minutes each. The slides were rinsed with two washes of dH_2O for 5 minutes each. Epitopes were revealed by boiling the slides in sodium citrate buffer (sodium citrate, pH 6.0) for 10 minutes. The slides were cooled in the buffer at least 30 minutes, rinsed in dH_2O three times for 5 minutes each, rinsed in PBS for 5 minutes and non-specific binding sites blocked with PBS containing 5% BSA for 60 minutes at 22°C. The slides were rinsed with PBS for 5 minutes, and then incubated with anti-E-cadherin antibody (BD Bioscience) overnight at 4°C. Slides were rinsed with three washes of PBS for 5

minutes each and then incubated with Alexa Fluor 488 donkey anti-mouse IgG (Invitrogen) for 60 minutes at 22°C. After further washing, cover slips were mounted on the slides and images were acquired using an Olympus (IX71) fluorescent microscope equipped with DPController Software (Olympus).

Hematoxylin and eosin (HE) staining of paraffin sections of fixed lung tissues

Paraffin was removed from sections by two washes of xylene for 10 min each. Slides were incubated in two washes of 100% ethanol for 10 minutes each, 95%, 85%, and 70% ethanol for 10 minutes each and rinsed with two washes of dH_2O for 5 minutes each. The slides were stained in Harris' hematoxylin solution for 3 minutes, then washed in running tap water for 5 minutes. The slides were differentiate in 1% acid alcohol for 30 seconds, then washed in running tap water for 1 minute. Slides were stained in 0.2% ammonia water for 30 seconds and washed in running water for 5 minutes. Slides were stained in eosin-phloxine solution for 2 minutes. Slides were dehydrate using 95% ethanol and cleared with 2 washes of xylene, 5 minutes each. Slides then had coverslips affixed to them.

Protein extraction and immunoblot analysis

Protein extraction and immunoblotting was carried out as previously described [5]. Briefly, protein samples for western blots were prepared by lysing cells in radioimmunoprecipitation assay (RIPA) buffer containing 1 mM EDTA. Lysates were separated by sodium dodecyl sulfate-polyacrylamide gel electrophoresis (SDS-PAGE), and transferred to polyvinylidene difluoride membranes. After transfer, the membranes

were then incubated for 1 h at 22°C in a dilution of a primary antibody in blocking solution containing 0.1% Tween-20. Next, the membranes were washed three times with PBS-Tween 20 and probed for 1 hour with the appropriate Alexa Fluor 680 (Molecular Probes) or IRdye 800 (Rockland Immunochemicals) conjugated secondary antibody. After three washes with PBS-Tween 20, blotted proteins were detected and quantified using an Odyssey infrared imaging system (LI-COR, Lincoln, Nebraska).

Flow cytometry

For cell surface marker staining, isolated cells were blocked for 5 min at 4°C with 10 µg/ml Mouse Fc Block (BD), and then reacted for 30 min at 4°C with various fluochrome-labeled Abs including appropriate isotype controls. After washing twice, cells were analyzed using a FACSCalibur (BD Biosciences).

Statistical analysis.

All data are presented as the mean ± SEM. Statistical analysis was determined using an unpaired Student *t* -test or one-way analysis of variance (ANOVA) followed by Bonferroni's post hoc test. A *p* value of less than 0.05 was considered statistically significant.

References:

1. Hajra KM, Chen DY-S, Fearon ER (2002) The SLUG Zinc-Finger Protein Represses E-Cadherin in Breast Cancer. Cancer Research 62: 1613-1618.
2. Chen C-Z, Li L, Lodish HF, Bartel DP (2004) MicroRNAs Modulate Hematopoietic Lineage Differentiation. Science 303: 83-86.
3. Xiaoyun Wu JKW, Hongmei Liu, Hongling Xiao, Robert Kralovics, Josef T. Prchal and John C. Kappes (2000) Development of a novel trans-lentiviral vector that affords predictable safety. Molecular Therapy 2.
4. Grizzle WE, Mountz JD, Yang PA, Xu X, Sun S, et al. (2002) BXD recombinant inbred mice represent a novel T cell-mediated immune response tumor model. Int J Cancer 101: 270-279.
5. Xiang X, Zhuang X, Ju S, Zhang S, Jiang H, et al. (2011) miR-155 promotes macroscopic tumor formation yet inhibits tumor dissemination from mammary fat pads to the lung by preventing EMT. Oncogene 30: 3440-3453.
