# Supplementary material for: Grhl2 Determines the Epithelial Phenotype of Breast Cancers and Promotes Tumor Progression
Source: PLoS One. 2012 Dec 17;7(12):e50781. doi: 10.1371/journal.pone.0050781 (PMC3524252; doi:10.1371/journal.pone.0050781)
Supplement: Table S1 — Genes significantly down- or up- regulated in disseminated 4T1 cells that have undergone EMT. Expression level was Log2 transformed. (PDF) [file pone.0050781.s009.pdf]

Table S1. Genes significantly down- or up- regulated in disseminated 4T1 cells that have undergone EMT. Expression level was Log2 transformed.

Gene Symbol	mRNA Accession	4T1-control cells recovered from primary tumors	4T1-control cells recovered from lungs, which have undergone EMT	4T1-Grhl2 cells recovered from lung
Krt7	NM_033073	11.04498	4.851732	11.24753
Epcam	NM_008532	9.430897	4.530786	9.222847
Lamc2	NM_008485	10.07374	5.626832	9.873795
Il24	NM_053095	9.89462	5.45419	9.424725
Esrp1	NM_194055	8.406046	4.355281	8.301373
Cldn7	NM_016887	8.94467	4.894601	9.260962
Krt19	NM_008471	9.53244	5.543701	9.164513
Cldn4	NM_009903	10.00734	6.146869	10.5487
Kcnk1	NM_008430	9.372211	5.613279	9.342992
Sfn	NM_018754	9.58915	5.961771	8.952487
Tmem54	NM_025452	9.165084	5.567413	9.802297
Krt14	NM_016958	9.113629	5.546437	9.19035
Wnt7a	NM_009527	9.002069	5.43549	8.555911
Mpzl2	NM_007962	7.8505	4.303717	8.065193
Lcp1	NM_008879	8.623374	5.191095	7.906635
Tacstd2	NM_020047	7.383813	4.134166	6.903043
Fermt1	NM_198029	8.248555	5.022185	8.12108
Rab25	NM_016899	8.245137	5.066799	8.503081
Cdh1	NM_009864	9.073104	5.981471	8.7114
Gjb3	NM_008126	8.858642	5.869986	8.858642
St14	NM_011176	8.667245	5.713353	8.315893
Macc1	ENSMUST00000048880	6.663526	3.728617	6.718266
Serpinb5	NM_009257	6.662786	3.736787	6.657445
Lad1	NM_133664	8.868539	6.086957	8.758102
Mal2	NM_178920	7.53006	4.77621	8.200264
Sfn	NM_018754	9.69515	6.962361	9.077053
Anxa8	NM_013473	8.330047	5.614426	8.48765
Ap1m2	NM_001110300	7.704555	5.058623	7.451653
Il1a	NM_010554	6.972837	4.362532	6.398082
Mmp9	NM_013599	9.122633	6.526849	8.681236
Jup	NM_010593	8.607635	6.165058	8.041181
Cldn3	NM_009902	8.845804	6.438354	8.702808
Sorbs2	NM_172752	7.36818	4.978859	6.973795
Perp	NM_022032	9.763565	7.396174	9.763565

Lama3	NM_010680	7.506166	5.164542	7.506166
Mapk13	NM_011950	8.687549	6.369659	8.034201
Itgb6	NM_001159564	6.793427	4.489787	6.73001
Serpinb2	NM_011111	6.995971	4.769957	6.030586
Mal	NM_010762	8.231762	6.079667	8.431036
Lcn2	NM_008491	7.454608	5.336023	7.500533
Moxd1	NM_021509	7.511811	5.399067	7.77447
Lamb3	NM_008484	8.743576	6.631112	8.824085
Rnu2	NR_004414	8.678821	6.567006	6.541327
Cxcr3	NM_009910	7.325644	5.236932	7.728054
Tmeff1	NM_021436	8.177303	6.10451	8.177303
Rnu2	NR_004414	8.998672	6.958988	6.818303
Rnu2	NR_004414	9.174925	7.135581	6.994556
Rnu2	NR_004414	9.174925	7.135581	6.994556
Rnu2	NR_004414	9.174925	7.135581	6.994556
Rnu2	NR_004414	9.174925	7.135581	6.994556
Lsr	NM_017405	8.087518	6.062011	7.976595
Fads2	NM_019699	9.467932	7.442813	9.043802
Ripk4	NM_023663	7.445794	5.510324	7.189949
Rnu2	NR_004414	8.669774	6.7426	6.695065
Anxa6	NM_013472	9.084456	7.161008	9.532501
Idi1	NM_145360	9.41051	7.488932	8.181924
Idi1	NM_145360	9.171036	7.315254	8.127873
Wbp5	NM_011712	9.606244	7.770895	8.104371
Cyb5	NM_025797	8.599787	6.774249	7.947563
Dsp	NM_023842	7.263672	5.454275	6.979595
Gipc2	NM_016867	6.6001	4.798093	7.019437
Ablim1	NM_178688	7.115868	5.320139	7.394264
Stard10	NM_019990	8.477548	6.682206	8.725239
Ptprf	NM_011213	7.636715	5.863556	7.289218
Gm10758	ENSMUST00000099340	8.265908	6.503192	7.827122
B3gnt3	NM_028189	8.163964	6.406206	8.163964
Tjp2	NM_011597	9.427662	7.688694	9.275223
Bhlhe40	NM_011498	8.260273	6.528247	7.603405
Gca	NM_145523	7.395787	5.666913	7.369709
Hook1	NM_030014	6.937346	5.212058	6.35425
Pdgfb	NM_011057	8.024346	6.302124	7.562928
Prss22	NM_133731	8.176064	6.458324	8.176064
Hist1h2bc	NM_023422	6.188893	4.475697	5.19059
B4galnt3	NM_198884	7.838023	6.135764	7.838023
Grhl2	NM_026496	7.225007	5.52408	7.225007

Bmp7	NM_007557	6.89686	5.197155	5.706387
Wnt7b	NM_009528	7.97201	6.274496	8.022828
Sh2d1b1	NM_012009	5.576422	3.917243	4.952527
Dsg2	NM_007883	6.332933	4.683637	6.332933
C130090K23Rik	BC016523	6.580921	4.965528	6.878836
Scd2	NM_009128	11.1946	9.582421	11.42393
Tnfaip3	NM_009397	9.056306	7.45541	8.822783
Gsn	NM_146120	9.615561	8.017839	9.477274
Npr3	NM_008728	8.205006	6.610063	8.391541
Galnt3	NM_015736	8.983625	7.397641	8.53065
Il18rap	NM_010553	8.194788	6.62954	8.513218
Ndufc2	NM_024220	9.921612	8.37548	8.866699
Inhba	NM_008380	8.62961	7.087011	9.026163
Pkp2	NM_026163	7.891384	6.361759	7.456557
Tmsb4x	NM_021278	9.780481	8.264164	8.714289
Pmepa1	NM_022995	11.25807	9.758192	10.79117
Adamts1	NM_009621	8.321311	6.82289	8.406101
Rn18s	NR_003278	14.05842	12.56258	11.59259
Atp2c2	NM_026922	7.085552	5.590291	8.069174
Irf6	NM_016851	7.804566	6.325281	7.266329
Ndufb6	NM_001033305	10.44991	8.977814	9.879602
Cxcl16	NM_023158	8.365502	6.906602	8.501619
Mfsd2	NM_029662	6.981534	5.554194	7.11114
Prl3d1	NM_008864	6.530017	5.111835	7.020853
Marveld2	NM_001038602	6.185907	4.767858	6.416134
Uchl3	NM_016723	9.633366	8.216647	8.819581
Gnpnat1	NM_019425	8.757491	7.34801	7.903356
Ctgf	NM_010217	10.81309	9.408682	10.80829
F2rl1	NM_007974	6.658391	5.271749	6.585497
Gnpnat1	NM_019425	8.700497	7.314655	7.862058
Serpinb9	NM_009256	7.004158	5.622816	6.860518
Prom2	NM_138750	6.25951	4.881881	6.320306
Snrpa1	NM_021336	10.03164	8.664556	9.314085
Ets2	NM_011809	9.37494	8.014639	9.28381
Itgb4	NM_001005608	7.876781	6.519966	7.805945
Fabp5	NM_010634	9.000153	7.643499	8.325874
Mcpt8	NM_008572	7.448786	6.105641	6.835718
Myl12b	NM_023402	11.33104	9.988542	10.08951
Msln	NM_018857	10.0003	8.659078	10.13239
Immp1l	NM_028260	8.321027	6.9844	7.734411
Ostf1	NM_017375	8.441702	7.10731	7.719178

Slc4a11	NM_001081162	7.040751	5.711068	7.061532
Ocln	NM_008756	6.059614	4.733363	6.059614
Prl3d1	NM_008864	6.416435	5.097682	6.82289
Tmc4	NM_181820	8.662731	7.3473	8.920373
Polr2k	NM_001039368	8.722582	7.410313	8.045219
Srp14	NM_009273	9.221395	7.909683	8.472539
Tslp	NM_021367	10.59201	9.281264	9.599719
Fads3	NM_021890	8.93508	7.625328	8.851822
Gadd45b	NM_008655	9.1698	7.876709	9.601107
Mrpl32	NM_029271	7.834432	6.550975	6.818437
Plk2	NM_152804	9.702341	8.426215	9.242146
Clu	NM_013492	10.30267	9.026934	10.22141
Naca	NM_001113199	11.29331	10.02215	10.14353
Tnk1	NM_031880	6.984676	5.714372	6.901084
Serpinb6b	NM_011454	8.002346	6.734603	7.687095
Thbs1	NM_011580	11.42043	10.15788	11.17784
Tmem102	NM_001033433	6.383982	5.133759	6.280475
Cmc1	NM_026442	8.898855	7.655158	8.209602
Has2	NM_008216	10.06673	8.824538	10.29095
Fabp5	NM_010634	8.866048	7.628134	8.300181
Lsm1	NM_026032	8.057519	6.820191	7.147842
Bace1	NM_011792	8.48456	7.251371	8.364769
Hsbp1	NM_024219	10.70329	9.47203	9.827971
Il23a	NM_031252	7.392425	6.165088	8.193972
Lxn	NM_016753	8.659699	7.436413	8.100895
Arpc3	NM_019824	8.62069	7.403537	7.855433
Chn1	NM_001113246	5.904076	4.693848	5.600596
Sap30	NM_021788	7.31012	6.102308	6.490685
Rny1	NR_004419	7.189942	5.985518	6.081221
Mpzl3	NM_176993	6.406575	5.204783	6.397399
Ndufs5	NM_001030274	8.382013	7.184865	7.700527
Magoh	NM_010760	10.57113	9.375073	10.13292
Rps27l	NM_026467	9.31644	8.12108	8.79115
Pigx	NM_024464	8.914721	7.719612	8.497587
Gng5	NM_010318	9.053978	7.862518	8.225494
Prl2c3	NM_011118	5.816799	4.629415	6.063755
Deb1	NM_026794	7.292165	6.105463	6.274496
Rpl27a	NM_011975	12.4242	11.24114	11.39078
Lsm5	NM_025520	7.797094	6.622222	6.744365
Ly6e	NM_008529	7.55622	6.381812	7.55622
Krt17	NM_010663	6.340071	5.166023	6.593575

Mgat4a	NM_173870	7.065282	5.893484	7.288134
Rps15a	NM_170669	10.04013	8.873004	8.873004
Apoo-ps	NR_004438	9.354549	8.187425	8.20285
Cisd1	NM_134007	9.032356	7.865238	8.479692
Erh	NM_007951	11.02522	9.868192	10.28171
Tnip3	NM_001001495	7.203763	6.047629	6.108699
Tmem184a	NM_001161548	6.57308	5.418463	6.587019
Prl3d3	NM_172156	4.96067	3.8067	5.243901
Dstn	NM_019771	10.73266	9.58079	10.0185
Med30	NM_027212	6.714962	5.566531	5.771198
Cks2	NM_025415	8.254196	7.111042	7.70015
Cgn	NM_001037711	7.162368	6.020663	7.140697
Slfn2	NM_011408	7.265718	6.125415	6.675452
Stmn1	NM_019641	10.85452	9.715339	10.36595
Stmn1	NM_019641	10.85221	9.71316	10.36535
Mrpl47	NM_029017	7.849764	6.712803	6.878948
Myo1d	NM_177390	6.179165	5.042562	6.165802
Brp44l	NM_018819	7.704672	6.568114	6.658391
Klk10	NM_133712	7.452419	6.317109	7.147635
Ier3	NM_133662	9.845216	8.710649	9.187384
Erh	NM_007951	11.02376	9.890688	10.35007
Mest	NM_008590	6.01137	4.878727	6.01137
Frg1	NM_013522	8.672813	7.542356	8.3792
Esrp2	NM_176838	6.957853	5.827573	6.906902
Rbm3	NM_016809	13.08579	11.9598	11.9598
Gvin1	NM_029000	4.440519	3.31798	4.331661
Tubb2a	NM_009450	7.067457	5.945007	6.399762
Lsm5	NM_025520	7.656744	6.536323	6.556332
Nuak1	NM_001004363	8.641809	7.521701	8.831636
Psma2	NM_008944	9.996403	8.876801	9.311278
Phlda2	NM_009434	8.096102	6.980229	7.991593
Siva1	NM_013929	8.526792	7.415466	7.842709
Cycs	NM_007808	11.39302	10.28441	10.64369
Siva1	NM_013929	8.132954	7.024991	7.84833
Zranb1	ENSMUST00000106157	6.824478	5.717396	5.717396
Card10	NM_130859	8.929484	7.822567	9.088261
LOC100048581	XR_034815	8.467574	7.361244	7.566716
Cstb	NM_007793	8.28659	7.181095	7.45812
Plau	NM_008873	9.444102	8.339906	9.500949
Dnajc19 // Dnajc19	NM_026332	10.21998	9.117007	9.583106

Ak3	NM_021299	8.126312	7.024151	8.059663
Erh	NM_007951	11.0705	9.968429	10.42663
BC002163	NR_002445	7.926003	6.826073	6.961347
Anxa1	NM_010730	12.49703	11.39869	12.29387
Ctla2a	NM_007796	7.492478	6.395082	6.425367
Ppp2r5c	NM_001135001	7.352935	6.255738	6.466141
Ccdc72	NM_183250	10.62454	9.528342	9.979794
Mrps10	BC096657	9.305479	8.209822	8.778822
Ctsc	NM_009982	7.480726	6.387498	7.069758
Enpp1	NM_008813	6.236821	5.145597	7.511515
Ngfrap1	NM_009750	10.20393	9.11289	9.532712
Fhl2	NM_010212	9.170063	8.087261	8.495106
Mt2	NM_008630	10.79718	9.717712	10.61707
Ldlr	NM_010700	10.18208	9.103199	10.03125
Anxa4	NM_013471	9.881767	8.806515	9.260216
Slain1	NM_198014	6.741637	5.668705	6.218652
Gdpd5	NM_201352	7.2628	6.191046	7.083347
Eif4a3	NM_138669	9.534084	8.467656	9.181368
Rps24	NM_011297	10.74346	9.677502	9.684996
Krt8	NM_031170	11.97989	10.91413	12.06263
Tomm7	NM_025394	9.308161	8.2442	8.418878
Pctk3	NM_008795	7.496755	6.433456	7.556818
Scin	NM_001146196	5.997383	4.935007	6.553371
Nox4	NM_015760	6.877326	5.815997	6.16045
Gm8095	XR_035704	6.856955	5.796204	6.107492
Spint1	NM_016907	8.511198	7.459078	8.54073
Dhcr24	NM_053272	9.559338	8.50772	9.535478
Ssbp1	NM_212468	9.94869	8.897883	8.971063
Plin2	NM_007408	10.52308	9.473078	10.11365
Cks2	NM_025415	7.824084	6.775413	7.242178
H2-Q6	NM_207648	8.517796	7.472862	8.253671
Ssu72	NM_026899	8.535717	7.490815	7.811295
C3	NM_009778	6.920574	5.878601	6.19402
Fuca2	NM_025799	8.197451	7.155639	8.521061
Hmgcs1	NM_145942	8.496953	7.457853	9.01763
Med28	NM_025895	8.475252	7.438701	8.189887
Tomm20	NM_024214	10.89032	9.854103	10.22724
Myc	NM_010849	10.98438	9.948965	10.32915
Irgm1	NM_008326	7.20425	6.170025	6.737278
Hist1h2ab	NM_175660	9.028379	7.996178	7.921321
Rpl27a	NM_011975	12.03306	11.00327	11.18189

Trrap	NM_001081362	8.512007	7.482305	7.563864
Fam167a	NM_177628	6.271897	5.244238	6.147593
Gpr126	NM_001002268	8.192831	7.165339	8.489888
Arhgef3	NM_027871	7.172869	6.145934	6.801212
Naca	NM_001113199	11.25977	10.23469	10.35866
Insig1	NM_153526	9.208999	8.184337	9.460687
Gm8936	XR_033047	7.033291	6.009153	6.031121
Stx8	NM_018768	7.632308	6.614977	6.777585
Gm5502	XR_032770	8.076011	7.061592	6.918782
Mmp10	NM_019471	7.910484	6.897303	7.147842
Tlr3	NM_126166	6.639335	5.627318	6.157365
Stoml2	NM_023231	9.788108	8.776511	8.981569
Mrps36	NM_025369	6.040849	5.030882	5.046224
Hist1h2bb	NM_175664	8.451344	7.442707	7.003909
Tomm20	NM_024214	10.76154	9.753506	10.11412
Txndc17	NM_026559	11.26284	10.25596	10.37966
Nlrc5	FJ889356	6.028334	5.023459	6.028334
Igsf9	NM_033608	7.018948	6.015075	7.304055
Ndrg1	NM_008681	8.413339	7.410118	8.436296
Serpine1	NM_008871	9.775796	8.774277	9.760303
Tpt1	NM_009429	12.79897	11.79786	12.18264
Glipr1	NM_028608	6.811392	5.810539	6.102542
Lpl	NM_008509	4.322881	5.326105	5.463435
Slc38a1	ENSMUST00000100262	8.63539	9.639384	9.523296
Chmp1a	NM_145606	7.098792	8.106498	8.209288
Arhgap29	NM_172525	6.842532	7.850421	6.71857
Dnahc6	ENSMUST00000114040	4.835418	5.85142	4.684231
Tiaf2	AF075717	7.143044	8.159895	7.44636
Ptges	NM_022415	8.201695	9.229635	7.805628
Tm4sf19	NM_001160402	4.939783	5.982142	6.358778
Slc16a13	NM_172371	8.433037	9.476974	8.707069
Dub2a	NM_001001559	11.19325	12.28543	12.14876
Olfr1372-ps1	BC055827	3.502699	4.606027	3.427535
Itgb7	NM_013566	7.608709	8.716471	7.575896
Lipa	NM_021460	6.975234	8.101502	7.585226
Itgax	NM_021334	4.088993	5.235608	5.235608
Otop1	NM_172709	8.030914	9.195772	7.7808
Slc5a3	NM_017391	9.575551	10.75661	10.08279
Unc13c	NM_001081153	5.995808	7.203105	6.222123
Ctss	NM_021281	6.005631	7.231945	8.484988

Laptm5	NM_010686	5.012343	6.264601	6.498451
Clec7a	NM_020008	5.016158	6.326968	7.122411
Loxl3	NM_013586	8.848949	10.16357	9.269601
Xdh	NM_011723	8.458717	9.816477	8.433337
Sele	NM_011345	7.512496	8.941723	7.512496
Gpr56	NM_018882	6.034778	7.512102	7.091874
Ccl3	NM_011337	4.286173	5.768861	6.86165
ATP6	ENSMUST00000119235	9.468039	10.99846	10.99846
Atp6v0d2	NM_175406	4.14625	5.812409	6.130073
Cd36	NM_001159557	3.940645	5.68541	6.243145
Ctsk	NM_007802	4.969829	6.87052	7.005441
LOC100044795	XM_001473515	6.426476	8.875941	8.588012
Cybb	NM_007807	4.369773	6.929111	7.422164
Lyz2	NM_017372	4.955112	7.783814	8.936812
Mmp12	NM_008605	6.218465	9.496169	9.972452
