# Supplementary material for: Grhl2 Determines the Epithelial Phenotype of Breast Cancers and Promotes Tumor Progression
Source: PLoS One. 2012 Dec 17;7(12):e50781. doi: 10.1371/journal.pone.0050781 (PMC3524252; doi:10.1371/journal.pone.0050781)
Supplement: Table S2 — Comparison of gene expression profiles among 4T1-control cells recovered from primary tumors and lungs, 4T1-Grhl2, 4T1-Wnt7A1 and 4T1-Wnt7A2 cells recovered from lungs. Transcription level was Log2 transformed. (PDF) [file pone.0050781.s010.pdf]

Table S2. Comparison of gene expression profiles among 4T1-control cells recovered from primary tumors and lungs, 4T1-Grhl2, 4T1-Wnt7A1 and 4T1-Wnt7A2 cells recovered from lungs. Transcription level was Log2 transformed.

Gene Symbol	mRNA Accession	4T1-control cells recovered from primary tumor	4T1-control cells recovered from lung, which have undergone EMT	4T1-Grhl2 cells recovered from lung	4T1-wnt7A1 cells recovered from lung	4T1-Wnt7A2 cells recovered from lung
Krt7	NM_033073	11.04498	4.851732	11.24753	12.57659	5.163532
Epcam	NM_008532	9.430897	4.530786	9.222847	10.27806	4.171853
Cldn4	NM_009903	10.00734	6.146869	10.5487	11.8092	6.819798
Cldn7	NM_016887	8.94467	4.894601	9.260962	10.52759	5.306964
Lamc2	NM_008485	10.07374	5.626832	9.873795	11.48107	6.409315
Tmem54	NM_025452	9.165084	5.567413	9.802297	10.43635	5.145865
Il24	NM_053095	9.89462	5.45419	9.424725	10.56086	5.685039
Esrp1	NM_194055	8.406046	4.355281	8.301373	9.659298	4.83378
Mpzl2	NM_007962	7.8505	4.303717	8.065193	9.642673	4.716056
Kcnk1	NM_008430	9.372211	5.613279	9.342992	10.9972	5.985833
Krt14	NM_016958	9.113629	5.546437	9.19035	10.02624	5.54759
Krt19	NM_008471	9.53244	5.543701	9.164513	10.24617	6.253193
Rab25	NM_016899	8.245137	5.066799	8.503081	10.16314	4.810693
Mal2	NM_178920	7.53006	4.77621	8.200264	10.18661	4.91008
Wnt7a	NM_009527	9.002069	5.43549	8.555911	10.21479	8.069146
Fermt1	NM_198029	8.248555	5.022185	8.12108	9.448018	4.887207
Sfn	NM_018754	9.58915	5.961771	8.952487	10.05426	5.675931
Macc1	ENSMUST00000048880	6.663526	3.728617	6.718266	8.123114	3.804957
Gjb3	NM_008126	8.858642	5.869986	8.858642	9.383731	6.043832
Serpinb5	NM_009257	6.662786	3.736787	6.657445	8.213003	3.952151
Anxa8	NM_013473	8.330047	5.614426	8.48765	9.551559	5.716373
Tacstd2	NM_020047	7.383813	4.134166	6.903043	8.222933	3.873209
Cdh1	NM_009864	9.073104	5.981471	8.7114	10.1185	5.994653
Lcp1	NM_008879	8.623374	5.191095	7.906635	8.734509	5.496056
Lad1	NM_133664	8.868539	6.086957	8.758102	9.617127	6.464608
St14	NM_011176	8.667245	5.713353	8.315893	9.974148	5.428282
Cxcr3	NM_009910	7.325644	5.236932	7.728054	9.012232	5.774617
Atp2c2	NM_026922	7.085552	5.590291	8.069174	9.086532	6.958585
Ap1m2	NM_001110300	7.704555	5.058623	7.451653	9.170959	5.33696
Moxd1	NM_021509	7.511811	5.399067	7.77447	8.502666	5.31145
Anxa6	NM_013472	9.084456	7.161008	9.532501	10.64333	8.643463
Perp	NM_022032	9.763565	7.396174	9.763565	11.1568	8.560097

Enpp1	NM_008813	6.236821	5.145597	7.511515	9.212892	5.147184
Mal	NM_010762	8.231762	6.079667	8.431036	9.91946	6.364203
Lama3	NM_010680	7.506166	5.164542	7.506166	8.252725	5.668349
Cldn3	NM_009902	8.845804	6.438354	8.702808	10.048	7.221225
Itgb6	NM_001159564	6.793427	4.489787	6.73001	8.379641	4.455852
Gipc2	NM_016867	6.6001	4.798093	7.019437	8.769955	4.395709
Lamb3	NM_008484	8.743576	6.631112	8.824085	10.01391	6.121684
Lcn2	NM_008491	7.454608	5.336023	7.500533	7.607965	6.760496
Mmp9	NM_013599	9.122633	6.526849	8.681236	9.415565	6.071103
Sfn	NM_018754	9.69515	6.962361	9.077053	10.01822	7.392962
Ablim1	NM_178688	7.115868	5.320139	7.394264	8.841002	6.127448
Tmeff1	NM_021436	8.177303	6.10451	8.177303	9.357855	7.278226
Stard10	NM_019990	8.477548	6.682206	8.725239	9.597331	7.197169
Il1a	NM_010554	6.972837	4.362532	6.398082	7.341865	4.887984
Il23a	NM_031252	7.392425	6.165088	8.193972	9.232384	5.939026
Sorbs2	NM_172752	7.36818	4.978859	6.973795	7.925342	5.171113
Ccl6	NM_009139	5.336937	6.056314	8.045918	8.447103	5.995707
Inhba	NM_008380	8.62961	7.087011	9.026163	10.29831	7.967233
Lsr	NM_017405	8.087518	6.062011	7.976595	9.308758	6.891533
C130090K23Rik	BC016523	6.580921	4.965528	6.878836	8.732874	4.857783
Prl3d1	NM_008864	6.530017	5.111835	7.020853	6.316263	5.489334
Il18rap	NM_010553	8.194788	6.62954	8.513218	8.520966	6.689131
Jup	NM_010593	8.607635	6.165058	8.041181	8.778715	6.699754
Scd2	NM_009128	11.1946	9.582421	11.42393	11.23856	10.48903
Npr3	NM_008728	8.205006	6.610063	8.391541	9.245607	7.261241
B3gnt3	NM_028189	8.163964	6.406206	8.163964	9.009356	6.911228
Wnt7b	NM_009528	7.97201	6.274496	8.022828	9.198164	7.34891
Prl3d1	NM_008864	6.416435	5.097682	6.82289	6.226648	5.331344
Gadd45b	NM_008655	9.1698	7.876709	9.601107	9.968818	7.982859
Prss22	NM_133731	8.176064	6.458324	8.176064	9.429991	6.837264
Gca	NM_145523	7.395787	5.666913	7.369709	8.643251	5.909497
B4galnt3	NM_198884	7.838023	6.135764	7.838023	9.471941	6.3741
Grhl2	NM_026496	7.225007	5.52408	7.225007	8.18425	5.968877
Ripk4	NM_023663	7.445794	5.510324	7.189949	8.663147	4.99991
Mapk13	NM_011950	8.687549	6.369659	8.034201	9.388186	7.049999
Dsg2	NM_007883	6.332933	4.683637	6.332933	7.434349	4.602347
Marveld2	NM_001038602	6.185907	4.767858	6.416134	7.289369	5.475246
Scin	NM_001146196	5.997383	4.935007	6.553371	7.556832	5.455566
Fads2	NM_019699	9.467932	7.442813	9.043802	9.533476	8.396692
Cxcl16	NM_023158	8.365502	6.906602	8.501619	9.165233	6.569018
Tjp2	NM_011597	9.427662	7.688694	9.275223	10.1702	8.121734

Adamts1	NM_009621	8.321311	6.82289	8.406101	9.619656	7.058831
Tmc4	NM_181820	8.662731	7.3473	8.920373	10.05118	8.824201
Hmgcs1	NM_145942	8.496953	7.457853	9.01763	9.087634	7.403842
Mfsd2	NM_029662	6.981534	5.554194	7.11114	7.916399	6.523466
Dsp	NM_023842	7.263672	5.454275	6.979595	8.006818	5.467061
Msln	NM_018857	10.0003	8.659078	10.13239	11.0216	8.94356
Has2	NM_008216	10.06673	8.824538	10.29095	10.23838	8.897199
Gsn	NM_146120	9.615561	8.017839	9.477274	10.2351	7.913645
Clec4n	NM_020001	3.422855	3.783646	5.238007	4.860714	3.988316
Prom2	NM_138750	6.25951	4.881881	6.320306	7.795797	4.966285
Krt17	NM_010663	6.340071	5.166023	6.593575	8.383623	5.513434
Ptprf	NM_011213	7.636715	5.863556	7.289218	8.119085	7.092372
Ctgf	NM_010217	10.81309	9.408682	10.80829	12.34735	9.73079
Mgat4a	NM_173870	7.065282	5.893484	7.288134	8.235008	5.395177
Sertad4	NM_198247	8.443526	7.514722	8.894011	8.53204	8.090016
Tnfaip3	NM_009397	9.056306	7.45541	8.822783	9.69298	7.621331
Fuca2	NM_025799	8.197451	7.155639	8.521061	9.076802	7.787577
Slc4a11	NM_001081162	7.040751	5.711068	7.061532	8.518899	6.556384
Ocln	NM_008756	6.059614	4.733363	6.059614	7.23863	4.541462
Gpr126	NM_001002268	8.192831	7.165339	8.489888	9.626497	7.824413
Gm10758	ENSMUST00000099340	8.265908	6.503192	7.827122	8.896812	6.689845
F2rl1	NM_007974	6.658391	5.271749	6.585497	7.408356	5.857488
Nuak1	NM_001004363	8.641809	7.521701	8.831636	9.840118	7.728471
Denr	NM_026603	9.612109	8.633605	9.938697	10.56894	8.808434
Fdft1	NM_010191	8.604467	7.752237	9.056544	9.267151	8.289154
Igsf9	NM_033608	7.018948	6.015075	7.304055	8.350577	5.986595
Itgb4	NM_001005608	7.876781	6.519966	7.805945	9.271418	7.0625
Mboat1	NM_153546	8.501143	7.506606	8.789145	9.606838	7.899465
Loxl4	NM_053083	8.28073	7.520425	8.799108	9.1124	7.926236
Insig1	NM_153526	9.208999	8.184337	9.460687	9.810321	8.281491
Ets2	NM_011809	9.37494	8.014639	9.28381	9.888217	8.359834
Arap2	NM_178407	6.674736	6.009547	7.276939	7.958915	5.118121
Card10	NM_130859	8.929484	7.822567	9.088261	9.941757	8.007938
Pdgfb	NM_011057	8.024346	6.302124	7.562928	7.866679	6.470087
Serpinb2	NM_011111	6.995971	4.769957	6.030586	7.014514	5.564525
Ctss	NM_021281	6.005631	7.231945	8.484988	8.971294	7.654566
Hsd17b7	NM_010476	8.16349	7.633814	8.884851	9.45185	7.958811
Serpinb9	NM_009256	7.004158	5.622816	6.860518	6.788998	5.763628
Fads3	NM_021890	8.93508	7.625328	8.851822	9.057448	8.797904
Itgb2	NM_008404	7.61349	6.741392	7.941715	8.514112	6.895793
Clu	NM_013492	10.30267	9.026934	10.22141	11.53061	9.849158

Mpzl3	NM_176993	6.406575	5.204783	6.397399	7.411163	5.19098
Tnk1	NM_031880	6.984676	5.714372	6.901084	7.529846	6.299747
Ly6e	NM_008529	7.55622	6.381812	7.55622	8.956602	7.149426
Cyb5	NM_025797	8.599787	6.774249	7.947563	7.86684	7.318111
Tmem184a	NM_001161548	6.57308	5.418463	6.587019	8.214695	5.613023
Plau	NM_008873	9.444102	8.339906	9.500949	10.46315	9.011817
Slc44a2	NM_152808	9.136899	8.381491	9.54191	9.831719	8.397217
Sc4mol	NM_025436	11.34377	10.3707	11.53035	11.54537	10.66812
Krt18	NM_010664	11.22217	10.28611	11.44295	12.14541	10.37173
Lyz2	NM_017372	4.955112	7.783814	8.936812	9.574326	7.81194
Krt8	NM_031170	11.97989	10.91413	12.06263	12.74016	11.23415
Ifi27l1	NM_026790	9.006354	8.042812	9.19035	9.654258	8.741509
Tmem102	NM_001033433	6.383982	5.133759	6.280475	6.867692	6.280475
Hook1	NM_030014	6.937346	5.212058	6.35425	7.796865	6.717051
Galnt3	NM_015736	8.983625	7.397641	8.53065	10.15347	7.634485
Mest	NM_008590	6.01137	4.878727	6.01137	8.137255	5.517141
Pctk3	NM_008795	7.496755	6.433456	7.556818	8.222527	6.892017
Myo1d	NM_177390	6.179165	5.042562	6.165802	7.265519	6.089352
Cgn	NM_001037711	7.162368	6.020663	7.140697	8.670987	6.919716
Bace1	NM_011792	8.48456	7.251371	8.364769	9.310427	7.217875
Adamts6	NM_001081020	6.498451	5.635218	6.74054	7.336395	6.27908
Rdh10	NM_133832	9.682044	8.922714	10.02403	10.91707	8.888531
Pkp2	NM_026163	7.891384	6.361759	7.456557	8.144812	6.473478
Ccl3	NM_011337	4.286173	5.768861	6.86165	6.783302	5.35526
Spint1	NM_016907	8.511198	7.459078	8.54073	9.596301	7.973344
Esrp2	NM_176838	6.957853	5.827573	6.906902	7.893761	6.294188
Bhlhe40	NM_011498	8.260273	6.528247	7.603405	8.254091	6.909166
Tnnt2	NM_001130174	10.66118	9.857195	10.92077	11.10161	10.27123
Paox	NM_153783	8.327385	7.433562	8.487122	9.822964	7.120106
Ak3	NM_021299	8.126312	7.024151	8.059663	8.28719	8.059663
Sh2d1b1	NM_012009	5.576422	3.917243	4.952527	6.789133	3.965419
Pmepa1	NM_022995	11.25807	9.758192	10.79117	11.32411	10.03235
Rtp4	NM_023386	6.125906	5.202027	6.234931	6.68004	5.179189
Dhcr24	NM_053272	9.559338	8.50772	9.535478	10.83929	9.033998
Ndrg1	NM_008681	8.413339	7.410118	8.436296	9.378439	8.142682
Fam83a	NM_173862	6.283362	5.942109	6.967381	7.133237	6.06656
Thbs1	NM_011580	11.42043	10.15788	11.17784	11.7871	10.87393
Gvin1	NM_029000	4.440519	3.31798	4.331661	5.588289	4.128791
Loxl4	BC053925	8.04775	7.083346	8.096605	8.666151	7.245265
Phlda2	NM_009434	8.096102	6.980229	7.991593	9.10314	7.173739
Nlrc5	FJ889356	6.028334	5.023459	6.028334	6.769257	5.771198

Pcyt1b	NM_211138	8.639697	7.788961	8.792505	9.133613	8.639697
Tspan2	NM_027533	7.149976	7.353631	8.347898	8.827682	7.756082
Fam169a	NM_001100458	6.18419	5.261137	6.253021	7.312213	5.08962
Serpine1	NM_008871	9.775796	8.774277	9.760303	10.23088	9.295989
Ank3	NM_146005	6.396924	5.414009	6.396924	7.142051	6.19972
Ddit4	NM_029083	6.917621	6.361274	7.342527	7.804857	6.680386
Acta2	NM_007392	5.594308	5.467428	6.446464	7.127413	5.297952
Cyp51	NM_020010	8.498299	7.992376	8.970617	9.323673	8.216322
Cd82	NM_007656	9.543251	9.226689	10.19648	10.83654	9.517932
Endod1	NM_028013	9.30636	8.570078	9.535381	10.6187	8.283609
Fdft1	NM_010191	9.693757	8.925359	9.882765	10.28929	9.423352
Trim13	NM_023233	5.458918	4.533463	5.488821	5.535086	5.427627
Fgd3	NM_015759	7.33818	6.383509	7.33818	7.798872	6.713443
Col7a1	NM_007738	7.810647	7.066897	8.017603	8.712323	7.469141
Nuak2	NM_028778	7.630949	6.820615	7.769412	8.051155	6.69823
Irf6	NM_016851	7.804566	6.325281	7.266329	7.939065	6.921171
Prnd	NM_023043	5.657236	4.666655	5.605775	7.482859	4.528136
Grb7	NM_010346	7.065516	6.126678	7.065516	8.057337	5.35296
Sqle	NM_009270	9.285295	8.682278	9.620715	9.843607	8.678938
Ldlr	NM_010700	10.18208	9.103199	10.03125	10.4385	9.680424
Tspan14	NM_145928	9.452105	8.952911	9.880286	10.28394	9.428045
Pthlh	NM_008970	7.036569	6.692268	7.607666	7.85473	7.069488
Slc9a3r1	NM_012030	8.493892	7.543667	8.454556	8.918639	7.226585
Chn1	NM_001113246	5.904076	4.693848	5.600596	6.332933	5.764506
Fam167a	NM_177628	6.271897	5.244238	6.147593	7.137544	5.202902
Sema4d	NM_013660	7.00913	6.123701	7.024097	7.802321	6.313807
Mt2	NM_008630	10.79718	9.717712	10.61707	11.26434	10.2955
Klk8	NM_008940	6.087296	5.190807	6.087296	7.425677	5.410378
Anxa1	NM_010730	12.49703	11.39869	12.29387	13.01756	11.51841
Gdpd5	NM_201352	7.2628	6.191046	7.083347	7.88882	6.419894
Cyp2j6	NM_010008	4.745035	3.853044	4.745035	6.23427	4.745035
Gm10777	ENSMUST00000099432	4.890294	4.858094	5.746465	5.942157	5.393085
Lss	NM_146006	7.795219	7.52359	8.411433	8.582477	8.010828
Tmprss11e	NM_172880	10.67852	10.06187	10.94701	11.48206	10.76375
Rasef	NM_001017427	5.302313	4.418278	5.302313	7.287017	4.340646
Acsbg1	NM_053178	8.58533	7.712662	8.58533	9.216291	7.634473
Sdc3	NM_011520	6.67476	6.151963	7.024126	7.364682	7.090822
Spry1	NM_011896	7.225944	6.354198	7.225944	8.385924	7.083347
Htr5b	NM_010483	6.296688	5.42921	6.296688	8.138699	5.718519
Zdhhc23	NM_001007460	6.822194	5.918363	6.771957	8.488909	6.314644
S100a14	NM_025393	4.92794	4.255307	5.102008	6.237563	4.806145

Gm10661	ENSMUST00000098707	6.975032	6.87052	7.712723	8.530625	7.639299
Mtap7	NM_008635	6.559792	5.735215	6.568745	7.797123	6.275
Gpa33	NM_021610	9.971588	9.097013	9.930079	10.58981	9.703203
Klk10	NM_133712	7.452419	6.317109	7.147635	7.528833	7.229129
Serpina1c	NM_009245	3.243855	2.770093	3.594117	3.95876	3.808916
Adam12	NM_007400	7.201829	6.393662	7.217059	8.275255	6.423334
Cda	NM_028176	7.157264	6.335509	7.157264	8.049278	6.807517
Plk2	NM_152804	9.702341	8.426215	9.242146	9.790659	7.908919
Tc2n	NM_028924	3.79463	2.833122	3.648539	4.273728	3.388692
Cdkl2	NM_016912	6.561194	5.943191	6.754941	7.425357	6.532092
Nipal2	NM_145469	8.527951	7.651409	8.461616	9.742907	8.340217
Csf3	NM_009971	6.922569	6.107649	6.917424	7.942843	6.616383
Itgam	NM_001082960	3.90037	4.419797	5.229078	5.658122	4.803478
St3gal6	NM_018784	6.717562	5.847891	6.651906	7.598822	6.35288
Hmgcr	NM_008255	9.261324	8.68907	9.492918	9.738096	8.839516
Sphk1	NM_011451	7.293815	6.500422	7.303366	8.083027	7.0807
Tgm2	NM_009373	6.871353	6.361618	7.161236	8.641918	6.430066
Htatip2	NM_001146049	6.774379	6.478713	7.277577	7.913926	6.837439
Car9	NM_139305	6.018203	5.222598	6.018203	8.069405	5.160692
Kank3	NM_030697	7.066224	6.191497	6.974878	7.696188	6.129848
Gcnt2	NM_023887	7.395986	6.631702	7.414258	8.100353	7.395986
Rnf128	NM_023270	7.089445	6.211835	6.990873	7.898526	7.059406
Pigx	NM_024464	8.914721	7.719612	8.497587	8.932559	7.431573
Tmem40	NM_144805	7.383813	7.196609	7.974174	8.731498	6.604226
Entpd3	NM_178676	7.01859	6.308293	7.084971	8.702713	6.470494
Il18r1	NM_008365	3.871057	3.429857	4.20481	6.550867	3.752031
Ube2g2	NM_019803	8.977316	8.233948	8.995858	9.608709	8.717516
Lypd3	NM_133743	6.928519	6.168226	6.928519	8.218459	6.72053
Srebf2	NM_033218	8.428832	7.893263	8.649658	9.031162	8.22984
Cflar	NM_207653	7.69218	7.214138	7.96996	8.729033	7.793352
Tpd52	NM_001025261	6.108788	5.23353	5.985793	6.899864	4.941
Marveld3	NM_028584	5.827601	5.154214	5.905067	7.118781	5.504304
Rab32	NM_026405	6.840663	6.005366	6.750768	7.477456	6.335177
Tmbim4	NM_026617	8.764572	8.213547	8.954222	9.256173	8.671452
Mcpt8	NM_008572	7.448786	6.105641	6.835718	7.936418	6.258577
Rhoj	NM_023275	4.246883	4.058643	4.788661	5.796514	4.643104
Gm12528	ENSMUST00000107544	6.344617	5.618292	6.344617	7.565934	6.126014
Fam38b	NM_001039485	5.686621	4.817227	5.540943	5.912433	5.540943
Samd12	NM_177225	7.706064	6.982673	7.706064	9.165359	7.108961
Atp11a	NM_015804	9.459326	8.651094	9.370591	10.24399	9.366391

Gabarapl2	NM_026693	9.794588	9.305414	10.0161	10.45942	9.674719
Tom1l1	NM_028011	6.630527	5.921707	6.627163	7.544732	6.035902
Kdelr3	NM_134090	7.682742	7.193299	7.898258	8.326307	7.214534
Erbb3	NM_010153	5.687318	5.100474	5.800128	6.615807	5.111487
Cnnm4	NM_033570	8.274223	7.802089	8.497828	9.114859	7.832881
Elf3	NM_007921	5.833732	5.193683	5.883231	6.71305	5.392271
Csrp1	NM_007791	9.977863	9.100934	9.790215	10.21897	9.451444
Myo5c	NM_001081322	5.761274	4.99868	5.686902	6.664093	5.58048
Cd38	NM_007646	4.687772	4.726311	5.414009	6.0174	5.373193
Gvin1	NM_029000	4.188964	3.388797	4.076066	5.077028	3.87358
Ctsc	NM_009982	7.480726	6.387498	7.069758	7.51285	6.526914
Taf13	NM_025444	7.205904	6.975662	7.655442	8.001234	7.436969
Gjb4	NM_008127	7.382084	6.796561	7.476094	8.230539	6.705986
Atp13a4	NM_172613	5.565346	4.639649	5.316675	6.37183	4.798734
Usp18	NM_011909	5.603215	5.266956	5.941794	7.473421	5.085989
Cdcp1	NM_133974	8.819581	8.151841	8.819581	9.385478	8.489529
Wnt10a	NM_009518	6.872931	6.205595	6.872931	7.411427	6.85727
Ptafr	NM_001081211	5.209766	5.122534	5.783884	6.493303	5.503338
Sorcs2	NM_030889	6.48435	5.823321	6.48435	7.439535	6.230648
Sh3bp2	NM_001145859	6.920715	6.41522	7.07618	7.570001	6.417368
Rhob	NM_007483	7.972765	7.33471	7.992486	9.234525	7.914542
Arhgef3	NM_027871	7.172869	6.145934	6.801212	7.238562	6.250367
Pstpip2	NM_013831	5.373144	4.821648	5.475215	6.243726	4.770315
Gm9853	ENSMUST00000060171	8.32018	8.304996	8.958129	9.548351	8.872524
Prrg4	NM_178695	8.666559	7.771925	8.42487	8.894357	8.120008
Gsta4	NM_010357	7.750183	7.321989	7.972955	8.324379	7.404257
Cpox	NM_007757	8.586952	8.198752	8.849466	9.223432	8.586952
Cflar	NM_207653	7.74539	7.223246	7.87031	8.320809	7.554098
Lrrk2	NM_025730	4.11353	4.050993	4.693295	6.007985	4.189095
Sult2b1	NM_017465	6.658333	6.016739	6.658333	7.533799	6.280376
Plin2	NM_007408	10.52308	9.473078	10.11365	10.55512	9.710571
Tusc3	NM_030254	8.938357	8.237577	8.87396	9.293716	8.764876
Rgs12	NM_173402	7.882175	7.374764	8.008021	8.576152	7.713964
Fam83b	BC120577	5.069318	4.493721	5.11951	5.843514	4.815547
AU042651	NM_177809	6.294622	5.753907	6.368322	7.226529	6.009272
Carhsp1	NM_025821	9.013668	8.514961	9.119589	9.783624	9.100677
Sdcbp2	NM_145535	5.678521	5.081223	5.678521	6.443263	5.678521
Lif	NM_008501	7.703046	6.81956	7.4151	8.222567	6.791978
Sema7a	NM_011352	7.09069	6.501032	7.09069	8.587912	7.00423
B4galnt1	NM_008080	7.63236	7.042885	7.63236	8.611609	7.384382
Mtss1	NM_001146180	8.007401	7.612176	8.200847	8.695269	8.007401

P2ry2	NM_008773	6.190073	5.948435	6.534545	7.283912	5.536338
Plek2	NM_013738	6.050148	5.300328	5.885192	6.455051	5.207807
Nr2f6	NM_010150	7.664375	7.433661	8.017146	8.473351	7.84608
Itga2	NM_008396	9.913584	9.396119	9.977187	10.51946	9.588663
Rab21	NM_024454	10.15585	10.03571	10.61073	11.26839	10.17756
Mfsd6	NM_133829	7.27784	6.79007	7.364682	7.92963	7.297539
Ptpn22	NM_008979	7.770323	7.195813	7.770323	8.401474	7.770323
Irgm1	NM_008326	7.20425	6.170025	6.737278	7.336395	6.037442
Ankrd22	NM_024204	4.380218	4.514565	5.080181	5.5365	4.546692
Pkp3	NM_019762	7.96312	7.241289	7.804055	8.45501	7.558743
Car12	NM_178396	7.500811	6.939145	7.500811	8.170989	7.330705
Ifngr2	NM_008338	8.980139	8.581178	9.141875	9.583388	8.500804
Mthfd2l	NM_026788	7.75602	7.230858	7.788544	8.301295	7.735061
Il17re	NM_145826	5.604266	5.235349	5.78385	6.570338	5.48288
Golim4	NM_175193	7.572598	7.108408	7.65611	8.623817	7.783981
Csnk1e	NM_013767	9.308582	9.116356	9.663581	10.3621	9.501366
Galc	NM_008079	7.313744	7.142207	7.687501	8.509676	7.161946
Spint2	NM_011464	10.83748	10.3422	10.88298	11.64144	10.66695
Cbr4	NM_145595	6.985937	6.154396	6.693725	7.201004	6.631539
OTTMUSG00000010657	NM_001083918	3.021716	2.988803	3.526576	4.768552	2.987697
Bspry	NM_138653	6.073545	5.804768	6.341942	7.157293	5.542693
Morc4	NM_029413	8.742199	8.018781	8.555054	9.509443	8.345998
Pttg1ip	NM_145925	10.4922	10.42198	10.9573	11.61766	10.48524
Gm8995	AK172683	5.563083	4.830901	5.361156	5.849994	4.577573
Tlr3	NM_126166	6.639335	5.627318	6.157365	7.132556	5.699521
Serinc5	NM_172588	7.307543	6.946146	7.474249	8.160053	7.435269
Krt80	NM_028770	7.106981	6.58126	7.106981	7.775955	6.545545
F3	NM_010171	5.681891	4.889037	5.414009	6.062547	4.955402
Rnf152	NM_178779	5.337965	4.815819	5.337965	5.943692	5.337965
Parp9	NM_030253	7.174429	6.8757	7.394333	7.888169	7.008682
Mt1	NM_013602	10.43939	9.489813	10.00711	10.5569	9.926108
Arhgef9	NM_001033329	4.903181	4.546787	5.062434	5.806445	4.565638
Myo5b	NM_201600	5.678521	5.163373	5.678521	6.473104	5.35827
Ppp1r2	NM_025800	7.710205	7.195688	7.710205	8.302765	7.710205
Nup210	NM_018815	7.101548	6.285843	6.799892	7.301415	6.942821
Irf9	NM_001159417	6.759789	6.226517	6.737411	7.238334	6.490343
Ano9	NM_178381	5.393577	4.993093	5.503671	6.707999	5.188525
Pgf	NM_008827	5.741953	5.774992	6.283515	7.452962	5.923419
Klf6	NM_011803	9.560075	8.6678	9.176222	10.02613	8.845781
Fam165b	NM_138743	8.827056	8.110679	8.616939	9.13217	8.235302
Inpp4b	NM_001024617	5.63507	5.058208	5.559265	6.452783	4.985951

Zdhhc17	BC051527	6.872908	7.199796	7.698116	8.281916	6.851886
Pard6b	NM_021409	8.088543	7.46281	7.958915	8.561306	7.480929
Erlin2	NM_153592	9.312089	9.045409	9.538857	10.07126	9.606182
Tmem56	NM_178936	4.175788	3.661932	4.1536	5.492835	4.047491
Myo1d	NM_177390	6.076957	5.587162	6.076957	6.608289	6.076957
Jkamp	NM_024205	8.343233	7.888139	8.375094	8.908714	8.206903
D1Ertd448e	ENSMUST00000097783	6.882133	7.016071	7.503026	8.179972	7.654955
Elovl7	NM_029001	8.030789	7.601493	8.087279	9.185453	8.001198
Cnksr1	NM_001081047	6.732662	6.244653	6.729144	7.565367	6.729144
Zhx2	NM_199449	6.670476	6.283022	6.76736	7.318431	6.034033
Cd40	NR_027852	6.077562	5.83126	6.315459	7.070314	6.114436
Aqp8	NM_007474	5.133784	4.650493	5.133784	6.810319	5.133784
Cck	NM_031161	4.981472	4.498761	4.981472	6.250193	4.909866
Trpv4	NM_022017	7.769257	7.22423	7.706043	8.245008	7.449649
Ggct	NM_026637	8.189552	8.057596	8.533501	9.414736	8.497102
Ano8	ENSMUST00000093450	7.744357	7.573263	8.046813	8.912252	7.884316
Plekhg3	NM_153804	7.546991	7.075762	7.546991	8.32842	7.205883
Myb	NM_010848	5.993464	5.522379	5.993464	7.05571	5.609909
S100a16	NM_026416	8.300181	7.831957	8.300181	9.030891	8.300181
Cpeb2	NM_175937	6.957469	6.254241	6.716017	7.449825	6.504754
Dennd2d	NM_001093754	5.190789	4.730173	5.190789	6.78201	4.896667
Ptprk	NM_008983	9.131063	8.670508	9.131063	9.853765	9.051515
Slc44a3	NM_145394	6.347761	5.965235	6.425492	7.529439	5.380041
Oasl1	NM_145209	6.73034	6.271733	6.73034	7.405014	6.488845
Faah	NM_010173	5.941374	5.424233	5.882437	7.002147	5.882437
Prss8	NM_133351	5.998567	5.717396	6.175147	6.815433	5.25206
Tmsb4x	NM_021278	9.780481	8.264164	8.714289	9.551597	7.257228
Myh14	NM_028021	6.400357	5.953645	6.400357	7.405183	6.059423
Lass3	DQ646881	5.009134	4.110262	4.556332	5.409376	4.533013
Mapkapk3	NM_178907	6.074718	5.636494	6.074718	6.713999	6.074718
Sptlc2	NM_011479	8.944968	8.585428	9.020272	9.760744	8.65244
Itga7	NM_008398	5.262719	5.3135	5.745635	6.471347	5.570635
Tpd52	NM_001025263	8.697382	8.406353	8.837136	9.798584	8.481112
Oas1g	NM_011852	5.627531	5.204783	5.627531	6.671122	5.146027
Sdc1	NM_011519	8.914885	8.374644	8.795755	9.94378	8.283167
Fstl3	NM_031380	8.314899	8.18323	8.604284	9.386096	8.189036
Rasgef1b	NM_145839	5.740843	4.863171	5.283734	6.03826	4.678569
Ttc9	NM_001033149	7.374946	6.694287	7.113442	7.818822	7.152403
Ppm1j	NM_027982	5.873152	5.496774	5.905532	6.805292	5.822844
Ephb2	NM_010142	7.078203	6.669772	7.078203	7.861774	6.879025

Tmem66	NM_026432	8.566902	8.244185	8.651826	9.257456	8.847048
Ube2v2	NM_023585	8.087529	8.103482	8.49747	9.394968	8.278699
Rem2	NM_080726	7.225568	6.824971	7.217744	7.897004	7.015834
Rpia	NM_009075	7.913258	7.156963	7.548251	8.468288	6.959362
Stxbp6	NM_144552	6.807517	6.416508	6.807517	7.465315	6.682433
Dmpk	NM_032418	8.469815	8.190581	8.579378	9.726621	8.756642
Nes	NM_016701	8.355672	8.0577	8.439098	9.64096	8.412006
Adssl1	NM_007421	5.860236	5.58483	5.962616	6.74636	6.489367
Omp	NM_011010	5.543221	5.509612	5.883528	6.592048	5.635121
Rapgef5	NM_175930	3.862632	3.540347	3.914115	4.649215	3.862632
Gm5426	ENSMUST00000074615	5.011596	4.68844	5.059795	5.82082	5.059795
Cltb	NM_028870	9.601652	9.344213	9.709105	10.40378	9.709105
Atp8b1	NM_001001488	4.555839	4.083468	4.447853	5.487157	4.552223
Cmtm6	NM_026036	8.95789	8.602931	8.95789	9.830522	8.95789
Acp5	NM_001102404	8.235708	7.958751	8.313626	9.089215	8.197628
Chmp4c	NM_025519	6.513155	6.031387	6.371778	7.385172	5.877625
Csf2	NM_009969	4.902272	4.561964	4.902272	5.817967	4.710865
Arg1	NM_007482	4.84098	4.505696	4.84098	5.553898	4.84098
Ano1	NM_178642	8.240492	7.661918	7.994424	8.933771	7.828858
Vopp1	ENSMUST00000114297	7.628793	7.54974	7.877565	8.624293	7.988933
Cntfr	NM_016673	5.947714	5.566413	5.892478	6.763944	5.671778
Procr	NM_011171	8.669657	8.166529	8.476239	9.285238	8.061474
Rgs16	NM_011267	10.21535	9.978867	10.28488	11.12851	9.892798
Aspa	NM_023113	3.497351	3.195837	3.497351	4.575533	3.497351
Spns2	NM_153060	8.141314	8.147917	8.448296	9.439547	8.185534
Tbc1d30	NM_029057	5.275275	4.979863	5.275275	6.467856	5.275275
Mthfd2l	NM_026788	5.85287	6.434603	6.719903	7.616719	6.599874
Ereg	NM_007950	4.452437	4.170655	4.452437	5.488111	4.475273
Adm	NM_009627	5.294982	5.096863	5.371341	6.641149	5.294982
Mpv17l	NM_033564	5.772779	5.501456	5.772779	6.824031	5.724699
Arf2	NM_007477	9.225961	8.96739	9.225961	10.02937	9.351739
Mall	NM_145532	5.40917	5.257983	5.500131	6.829759	5.40917
Tmcc3	NM_172051	7.78388	7.546607	7.78388	8.678869	6.956626
Cbr2	NM_007621	5.770853	5.615652	5.842467	6.742022	5.842467
Sh3bgrl2	NM_172507	8.111501	7.70536	7.930892	8.967251	7.16151
Ptk2b	NM_001162365	7.296951	7.097617	7.296951	8.242077	6.957005
Sec22a	NM_133704	7.545619	7.355536	7.545619	8.392694	7.4123
Npnt	NM_033525	8.33047	8.229649	8.406046	9.356375	8.406046
Slc25a27	NM_028711	5.719414	5.66601	5.838789	6.670959	5.721689
Tle4	NM_011600	4.552328	4.342942	4.510422	5.346432	4.308558

Acox2	NM_053115	5.20009	4.914308	5.06824	5.972685	4.969075
Osgin1	NM_027950	6.982775	6.406206	6.547019	7.785167	6.345795
Ptplb	NM_023587	7.451315	7.318431	7.456239	8.415293	7.456239
Tnik	NM_026910	5.980592	5.911951	6.033861	7.619212	5.283587
Akr1c18	NM_134066	3.37382	3.275685	3.38865	4.309036	3.438124
Cpa4	NM_027926	4.759452	4.804852	4.909011	6.959333	4.817869
Grem1	NM_011824	5.107892	5.106635	5.20063	6.574838	5.212586
Ltf	NM_008522	4.460732	4.695578	4.78876	6.961106	4.78876
Ifna12	NM_177361	3.086471	2.615657	2.700888	3.996581	2.765882
Vav1	NM_011691	4.98859	4.907313	4.98859	7.026591	4.639789
Rcan1	NM_001081549	7.267081	6.793566	6.841765	7.977551	7.056077
Gng11	NM_025331	4.370418	4.370418	4.370418	5.647369	4.007097
Tspan1	NM_133681	4.250128	4.452437	4.452437	5.581511	4.452437
Parvb	NM_133167	4.663287	4.942589	4.942589	6.035735	4.920199
V1rh10	NM_134235	2.66473	2.720166	2.711428	3.865432	2.586626
Gzme	NM_010373	4.621649	4.621649	4.577573	6.123673	4.621649
Ssxb5	NM_199319	2.665405	2.756547	2.709426	3.757854	3.165953
Tnc	NM_011607	6.672742	6.416606	6.335017	8.15132	6.752571
Sftpc	NM_011359	4.584285	4.848815	4.577998	8.442349	4.848815
Olfr172	NM_147001	3.64638	3.286124	2.911606	4.612188	2.957394
Itga8	NM_001001309	5.857696	5.711968	5.309551	6.739483	6.333477
