# Supplementary material for: Grhl2 Determines the Epithelial Phenotype of Breast Cancers and Promotes Tumor Progression
Source: PLoS One. 2012 Dec 17;7(12):e50781. doi: 10.1371/journal.pone.0050781 (PMC3524252; doi:10.1371/journal.pone.0050781)
Supplement: Table S3 — Primers used for Realtime PCR and RT-PCR. (PDF) [file pone.0050781.s011.pdf]

Table S3. Primers used for Realtime PCR and RT-PCR

Gene name	Primers	Product length
Mouse Epcam	GAGTCCCTGTTCCATTCTT	250 bp
	TCTCCTTTATCTCAGCCTTC	
Mouse E-Cadherin	GTCTCCTCATGGCTTTGC	228 bp
	CTTTAGATGCCGCTTCAC	
Mouse Twist1	AGCGGGTCATGGCTAACG	162 bp
	GGACCTGGTACAGGAAGTCGA	
mouse pri-miR-141	GGTCAGTTCCTTCAGCAGTG	185 bp
	CCAAATGTGTTTCCAAATCT	
mouse pri-miR-200a	CCGAAGACAGGCTGATGATA	201
	GTGACAATCCACTTCTACTC	
mouse pri-miR-429	ACTGCCCTGCTGATGGATGT	170 bp
	ACAGTTGTAACAGATGGATT	
mouse pri-miR-200c	TGATCTTGAAGGTGGACTGG	284 bp
	CACTGGATTGGAGGAGGG	
Mouse Zeb2	GAGCTTGACCACCGACTC	223 bp
	TTGCAGGACTGCCTTGAT	
mouse pri-miR-200b	tgcagggctttctgctgttgtcc	
	cacccatatcgccaaggctaact	
Mouse Foxc2	TCGACCCGGACTCCTACAA	255 bp
	GGCTCAGCGTCTCCACCTT	
mouse beta-actin	CTGTCCCTGTATGCCTCTG	218 bp
	ATGTCACGCACGATTTCC	
Mouse DSG2	ACGGGCAGATAACAGACA	273 bp
	GGGTCACAATCCCTTCAT	
Mouse DSP	GCAACAAGCCCATTATCC	203 bp
	GCAAGAAAGGTCCACAGC	
Mouse Pkp2	GCAGCCGAAGTAGGAAAC	216 bp
	CCACCAATGTGGGTATCG	
Mouse Cldn3	GGCGGCTCTGCTCACCTTA	116 bp
	CGTACAACCCAGCTCCCATC	
Mouse cldn4	GGGAATCTCCTTGGCAGTC	190
	GGCGAGCATCGAGTCGTA	
Mouse Grhl2	TTTGGTCCAACACCGTCTA	162 bp
	CACTGGCAGCCCATACTT	
Mouse GAPDH	GTTGTCTCCTGCGACTTCA	184 bp
	GGTGGTCCAGGGTTTCTTA	
Mouse cldn7	GGGAGATGACAAAGCGAAGA	208 bp
	CAGAAGGACCAGAGCAGACC	

Mouse Gjb3	AGCATCGCCAGAAGCACG<br>GGCAGAAGCGCCTACCAT	278 bp
Mouse ocln	ATCCACCTATCACTTCAGA<br>TAATCTCCCACCATCCTC	292 bp
Mouse Tjp2	AAGTTCCCTGCCTACGAG<br>ATTCAACCGAACCACTCC	189 bp
Mouse Wnt7A	GGCAACCTGAGCGACTGT<br>AGTAATTGGGTGACTTCTCG	457 bp or 302 bp
Mouse Snai1	GTCGTCCTTCTCGTCCACC<br>GGCCTGGCACTGGTATCTC	391 bp
Mouse Snai2	TCCCATTAGTGACGAAGA<br>CCCAGGCTCACATATTCC	204 bp
Mouse Axin2	AGCCTAAAGGTCTTATGTGG<br>ATGGAATCGTCGGTCAGT	287 bp
Mouse Esrp1	TATTGAATCACCAGGGACG<br>TTCGGTAGGAATCACAGC	269 bp or 257 bp
Mouse CD44	GAAGGGCGAGTATAGAAC<br>AGATGCCAAGATGATGAG	To detect alternative splicing
Mouse CTNND1	GAACGAGGAAGAGGAAGA<br>GTACAGAAGGTGGTTGTGAT	612 bp, 494 bp, 205 bp
Mouse Enah	AGGTGGAGGGAATACTGG<br>GCACTTGGCTGTGATGAA	333 bp, 396 bp
Mouse MAP3K7	CTGCCACAAACGACACTA<br>GTCCCAGTAACAGTCAAGTC	328 bp, 247 bp
Mouse ID2	GGTGGACGACCCGATGAGT<br>TGCCTGCAAGGACAGGATG	268 bp
Mouse Zeb1	CCATACGAATGCCCGAACT<br>ACAACGGCTTGCACCACA	386 bp
Mouse TCF4	CACAACGGAGCGATGGGTA<br>GGGTGGGTTCAAGTCAGG	195 bp
Human Wnt7A	AGGGCAACCTGAGCGACTG<br>GGGCGTACTGGTGGGTGTT	585 bp
Human beta Actin	CACCAACTGGGACGACAT<br>ACAGCCTGGATAGCAACG	189 bp
Human GAPDH	GGATTTGGTCGTATTGGG<br>GGAAGATGGTGATGGGATT	205 bp
Human E-Cadherin	CTGAGAACGAGGCTAACG<br>TTCACATCCAGCACATCC	289 bp
Human CD24	TACCCACGCAGATTTATT<br>AGAGTGAGACCACGAAGA	162 bp
Human Epcam	ATCGTCAATGCCAGTGTA	288 bp

	CTCGCTCAGAGCAGGTTA	
Human Grhl2	CTTGACATTGCCGATTAC	224 bp
	ATGGGTTTATTGCTACGATT	
Human pri-miR-200a	CTTACCGGACAGTGCTGGAT	72 bp
	GCGGGTCACCTTTGAACATC	
Human pri-miR-200b	AGCTTCCCAGCGAGTCCCA	133 bp
	TCCGCCGTCATCATTACCAG	
Human pri-miR-200c	GTGTTTGGGTGCGGTTGG	83 bp
	AGGCGATGGATGTTGCTGAC	
Human pri-miR-141	CCACCCAGTGCGATTTGT	234 bp
	CGGGAGCCATCTTTACCAG	
Human vimentin	CCAGGCAAAGCAGGAGTC	213 bp
	CGAAGGTGACGAGCCATT	
Human Zeb1	GTGGCGGTAGATGGTAAT	254 bp
	CTGTTTGTAGCGACTGGA	
Human Zeb2	ACCAGCCCTTTAGGAGTT	272 bp
	AGACCGACAGGCGGAATA	
Human Sani1	CCCCAATCGGAAGCCTAA	261 bp
	CCTTTCCCACTGTCCTCAT	
Human Snai2	TCCTGGTCAAGAAGCATT	274 bp
	GAGGAGGTGTCAGATGGA	
Human Twist1	CGGGAGTCCGCAGTCTTA	161 bp
	GCTTGAGGGTCTGAATCTTG	
