# Supplementary material for: Grhl2 Determines the Epithelial Phenotype of Breast Cancers and Promotes Tumor Progression
Source: PLoS One. 2012 Dec 17;7(12):e50781. doi: 10.1371/journal.pone.0050781 (PMC3524252; doi:10.1371/journal.pone.0050781)
Supplement: Table S4 — Primers used for constructing lentiviral and retroviral vectors. (PDF) [file pone.0050781.s012.pdf]

Table S4. Primers used for constructing lentiviral and retroviral vectors.

Name	sequence	description
mGrhl2 BamHI	GGATCC atgtcacaagagtcggacaa	for cloning of full length ORF of mouse Grhl2
mGrhl2 MluI	ACG CGT tcagatctccatcagcgtgatc	for cloning of full length ORF of mouse Grhl2
mus wnt7a BamHI	ggatcc **CGCCACC** atgacccggaaagcgcggcgc	For cloning of mouse Wnt7A ORF
muswnt7aR MluI	acg cgt tcacttgcacgtatacatctc	For cloning of mouse Wnt7A ORF
Mus Esrp1 BamHI	ggatcc atgacggcgtctccggattacttgg	For cloning of mouse Esrp1 ORF
Mus Esrp1 HindIII	acgcgt ttaaatacaaacccattctttgggt	For cloning of mouse Esrp1 ORF
hFirfly luc Sh1	tcgagcccGCCTGAAGTCTCTGATCAATTCAAGAGATTGATCAGAGACTTCAGGCTTTTTG	For constructing ShRNA retroviral vector targeting h firefly Luciferase.
hFirefly fluc Sh1 Re	aattcaaaaaGCCTGAAGTCTCTGATCAATCTCTTGAATTGATCAGAGACTTCAGGCgggc	For constructing ShRNA retroviral vector targeting *h* firefly Luciferase
hsGrhl2 Sh3	tcgagcccGGACAAAGCGAGTGCTCTTGTTTCAAGAGAACAAGAGCACTCGCTTTGTCCTTTTTG	For constructing ShRNA retroviral vector targeting human Grhl2
hsGrhl2 Sh3 Re	aattcaaaaaGGACAAAGCGAGTGCTCTTGTTCTCTTGAAACAAGAGCACTCGCTTTGTCCgggc	For constructing ShRNA retroviral vector targeting human Grhl2
