# Supplementary material for: Grhl2 Determines the Epithelial Phenotype of Breast Cancers and Promotes Tumor Progression
Source: PLoS One. 2012 Dec 17;7(12):e50781. doi: 10.1371/journal.pone.0050781 (PMC3524252; doi:10.1371/journal.pone.0050781)
Supplement: Figure S2 — (A) All probes representing 54,675 transcripts on Dataset GSE12777 were analyzed for differential gene expression between epithelial and mesenchymal cells and Pearson correlation coefficient r was calculated between each transcript and vimentin across all microarrays. Some of the transcripts that were highly expressed in epithelial cells and negatively co-regulated with vimentin are labeled in Red, and some of the transcripts that were highly expressed in mesenchymal cells and co-regulated with vimentin are labeled in Blue. (B) Microarray-based screening for epithelial-specific transcription factors co-regulated with E-cadherin in human breast cancer cells (Dataset GSE12777). Probes representing about 3,000 transcripts that were annotated as transcription factor, DNA binding protein, or RNA binding protein, were analyzed for differential expression between epithelial and mesenchymal cells and Pearson correlation coefficient r was calculated between each transcript and E-cadherin. (C) Molecular subtypes in dataset GSE12777 were identified by PAM305 breast cancer subtype gene classifier [6]. The results show that these cancer cells can be separated into three groups, a luminal group expressing luminal and epithelial markers, a basal A group expressing myoepithelial and epithelial markers, and a basal B group expressing mesenchymal markers. Grhl2 is expressed in both luminal group and basal A group. (D) Hierarchical clustering of 39 human breast tumor cells in Dataset GSE16795 based on expression levels of epithelial and mesenchymal markers. Grhl2 is expressed exclusively in epithelial cells (P = 3.91754E-08, two-tailed students t tests). (PDF) [file pone.0050781.s002.pdf]

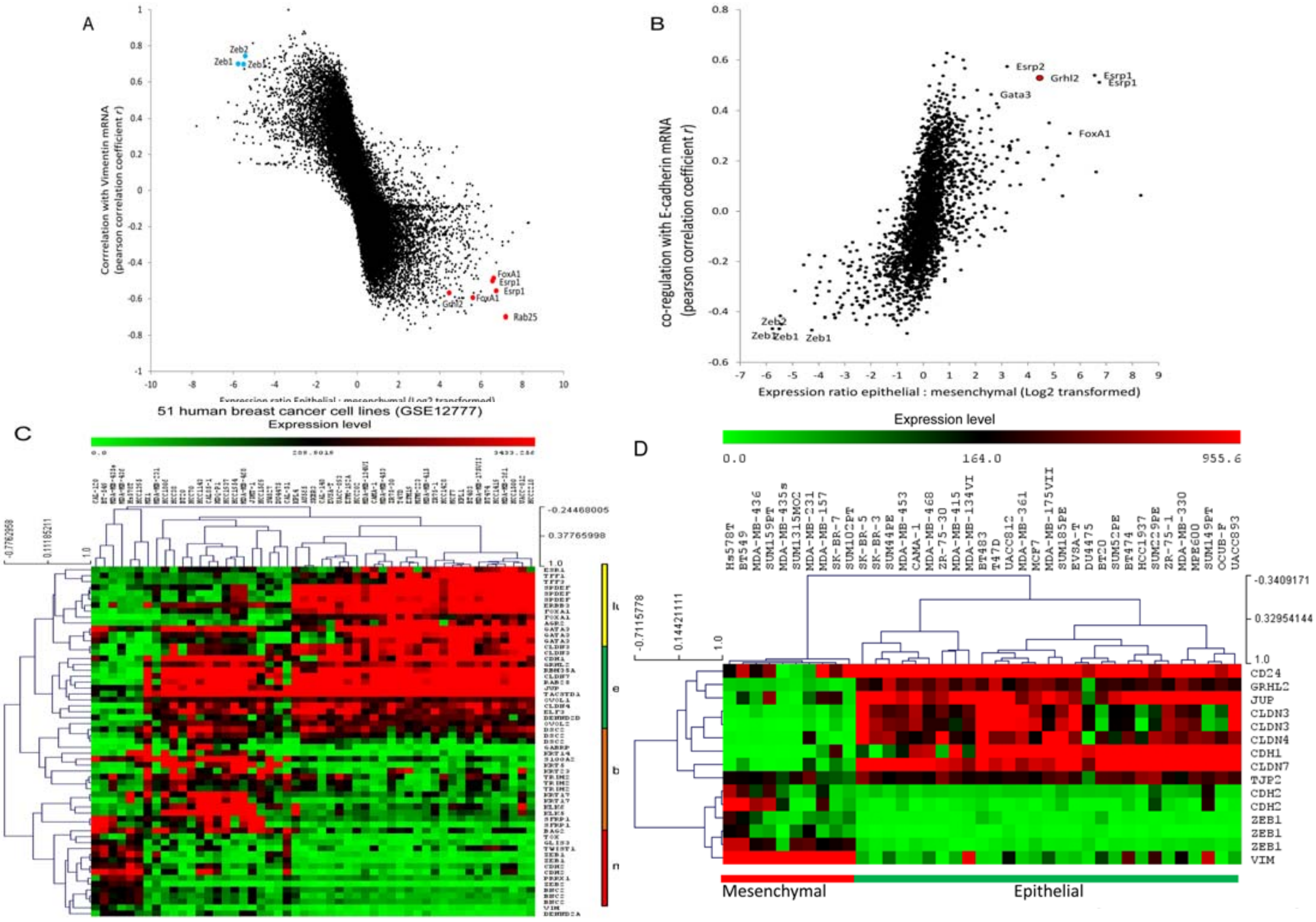

Figure S2

(A) All probes representing 54,675 transcripts on Dataset GSE12777 were analyzed for differential gene expression between epithelial and mesenchymal cells and Pearson correlation coefficient *r* was calculated between each transcript and vimentin across all microarrays. Some of the transcripts that were highly expressed in epithelial cells and negatively co-regulated with vimentin are labeled in Red, and some of the transcripts that were highly expressed in mesenchymal cells and co-regulated with vimentin are labeled in Blue. (B) Microarray-based screening for epithelial-specific transcription factors co-regulated with E-cadherin in human breast cancer cells (Dataset GSE12777). Probes representing about 3,000 transcripts that were annotated as transcription factor, DNA binding protein, or RNA binding protein, were analyzed for differential expression between epithelial and mesenchymal cells and Pearson correlation coefficient *r* was calculated between each transcript and E-cadherin.

(C) Molecular subtypes in dataset GSE12777 were identified by PAM305 breast cancer subtype gene classifier [1]. The results show that these cancer cells can be separated into three groups, a luminal group expressing luminal and epithelial markers, a basal A group expressing myoepithelial and epithelial markers, and a basal B group expressing mesenchymal markers. Grhl2 is expressed in both luminal group and basal A group.

(D) Hierarchical clustering of 39 human breast tumor cells in Dataset GSE16795 based on expression levels of epithelial and mesenchymal markers. *Grhl2* is expressed exclusively in epithelial cells (*P*=3.91754E-08, two-tailed students *t* tests).
