# Supplementary material for: Grhl2 Determines the Epithelial Phenotype of Breast Cancers and Promotes Tumor Progression
Source: PLoS One. 2012 Dec 17;7(12):e50781. doi: 10.1371/journal.pone.0050781 (PMC3524252; doi:10.1371/journal.pone.0050781)
Supplement: Figure S4 — Schematic representation of gene structure of two isoforms of mouse Wnt7A. (A)Two cDNA clones differing in about 155 base pairs were obtained by using specific primers to amplify the full length open reading frame of mouse Wnt7A from a cDNA pool derived from 4T1 cells. Sequencing results showed that the longer cDNA shared 100% sequence identity with the reference sequence of mouse Wnt7A (NM_009527.3), which encoded a protein 349 amino acids in length. This isoform is designated as Wnt7A isoform 1, or Wnt7A1. The shorter cDNA lacks 155 base pairs compared to the reference sequence of mouse Wnt7A, which is due to using an alternative 5′ splice site in the exon 3. This results in introducing an in-frame stop codon immediately after the alternative splicing site. This isoform encodes a truncated Wnt7A protein 148 amino acids in length. This isoform is designated as Wnt7A isoform 2 or Wnt7A2. (B) Schematics represent two alternative splicing sites that generate Wnt7A1 and Wnt7A2. Mouse Wnt7A exon3 is labeled in red and green colors, and adjacent introns were labeled in black colors. The sequence excluded in Wnt7A2 is labeled in green color. Two splicing sites are underline labeled. These two splicing sites share a sequence similarity. (C) Wnt7A1 and Wnt7A2 were introduced into 4T1 cells by lentiviral infection. Gene expressions in 4T1-control, 4T1-Wnt7A1 and 4T1-Wnt7A2 cells were analyzed by quantitative realtime PCR. (PDF) [file pone.0050781.s004.pdf]

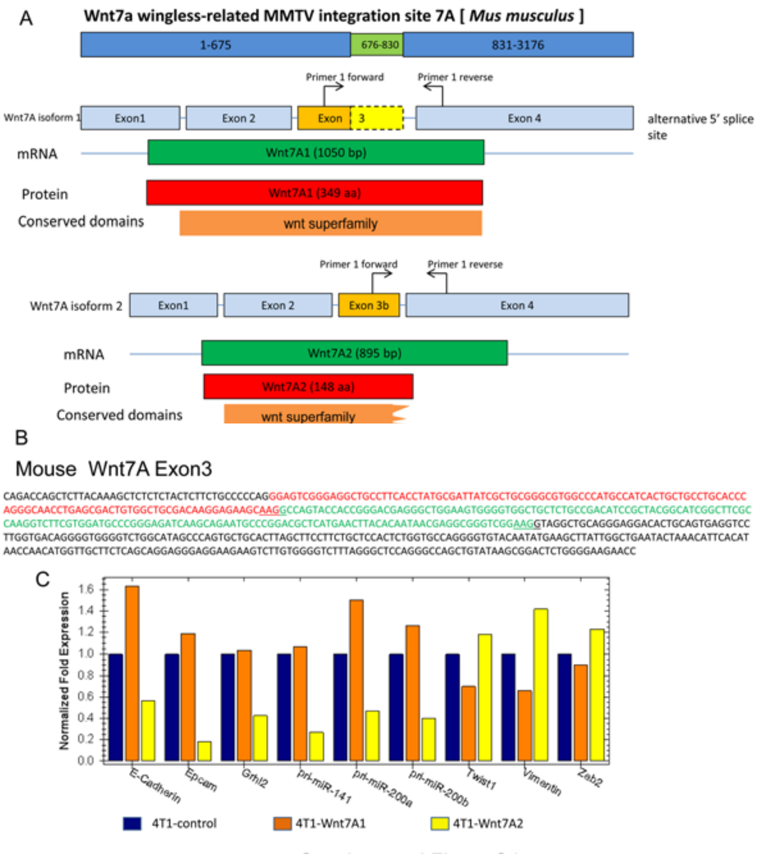

Figure S4 Schematic representation of gene structure of two isoforms of mouse Wnt7A. (A)Two cDNA clones differing in about 155 base pairs were obtained by using specific primers to amplify the full length open reading frame of mouse Wnt7A from a cDNA pool derived from 4T1 cells. Sequencing results showed that the longer cDNA shared 100% sequence identity with the reference sequence of mouse Wnt7A (NM_009527.3), which encoded a protein 349 amino acids in length. This isoform is designated as Wnt7A isoform 1, or Wnt7A1. The shorter cDNA lacks 155 base pairs compared to the reference sequence of mouse Wnt7A, which is due to using an alternative 5' splice site in the exon 3. This results in introducing an in-frame stop codon immediately after the alternative splicing site. This isoform encodes a truncated Wnt7A protein 148 amino acids in length. This isoform is designated as Wnt7A isoform 2 or Wnt7A2. (B) Schematics represent two alternative splicing sites that generate Wnt7A1 and Wnt7A2. Mouse Wnt7A exon3 is labeled in red and green colors, and adjacent introns were labeled in black colors. The sequence excluded in Wnt7A2 is labeled in green color. Two splicing sites are underline labeled. These two splicing sites share a sequence similarity. (C) Wnt7A1 and Wnt7A2 were introduced into 4T1 cells by lentiviral infection. Gene expressions in 4T1-control, 4T1-Wnt7A1 and 4T1-Wnt7A2 cells were analyzed by quantitative realtime PCR.
